# Unraveling the HIV-malaria interactions: a bibliometric analysis of global research trends and emerging insights

**DOI:** 10.3389/fmicb.2025.1622769

**Published:** 2025-08-29

**Authors:** Huize Zhang, Guoren Chen, Ping Ma, Yu Lai

**Affiliations:** School of Basic Medicine, Chengdu University of Traditional Chinese Medicine, Chengdu, China

**Keywords:** HIV, malaria, co-infection, interactions, bibliometric analysis, visualized analysis

## Abstract

**Background:**

The co-infection of human immunodeficiency virus (HIV) and malaria has emerged as an urgent public health challenge in tropical areas where the two diseases geographically converge, stimulating extensive biomedical investigations into their pathobiological interactions. This study aims to elucidate the current status and research trends regarding the interactions between HIV and malaria through bibliometric and visualized analysis.

**Methods:**

Research on the interactions between HIV and malaria was collected from the Web of Science Core Collection. Bibliometric analysis and knowledge graph visualization were performed on 4,717 articles published between 1995 and 2024, using CiteSpace software.

**Results:**

The results demonstrate a fluctuating upward pattern in the number of publications related to HIV and malaria interactions. According to the cooperative network visualization analysis, the United States, the University of London, and Grant Dorsey possess the greatest publication counts among all countries, institutions, and authors, respectively. The keyword and cocited reference analysis distinguish the primary research hotspots and frontiers as the epidemiological study of different populations in the African region, pathogenic mechanisms underlying HIV-malaria co-infection, strategies for the prevention and treatment of HIV and malaria co-infection, interactions between antimalarial and antiretroviral drugs, and malaria vaccine responses in HIV-infected individuals.

**Conclusion:**

This bibliometric investigation outlines the research hotspots, frontiers, and trends regarding the interactions between HIV and malaria. Future research should delineate pharmacokinetic and pharmacodynamic interactions between antimalarial and antiretroviral drugs to enhance clinical efficacy and medication safety and develop effective malaria vaccines that benefit HIV-positive populations in endemic areas.

## 1 Introduction

Human immunodeficiency virus (HIV) and *Plasmodium* infection are currently classified among the most prominent global health issues. HIV has led to an estimated 42.3 million deaths since the start of the epidemic, and by the end of 2023, approximately 39.9 million individuals were living with the virus worldwide (WHO, 2024a). In 2023, 83 malaria-endemic countries reported nearly 263 million estimated malaria cases, an increase of 11 million over 2022, culminating in about 597,000 deaths (WHO, 2024c). More importantly, the geographic spread of *Plasmodium* and HIV infections substantially overlaps in tropical regions, particularly in sub-Saharan Africa, thereby presenting a multifaceted global health challenge. Consequently, the dual infection in sub-Saharan Africa augments the prevalence and severity of clinical malaria, increases the likelihood of placental malaria during pregnancy, reduces the efficacy of malaria treatment, and expedites the progression of HIV to acquired immunodeficiency syndrome (AIDS) (Kwenti, 2018). These interactions strain healthcare systems and disproportionately affect vulnerable populations, particularly in resource-limited tropical regions. Understanding the complex dynamics of HIV and malaria co-infection is essential to developing integrated therapeutic and preventive strategies, improving clinical outcomes, and addressing this pressing global health challenge.

Bibliometrics is the quantitative analysis of academic publishing, focusing on using statistical methods to describe trends and relationships within the body of published works (Ninkov et al., 2022). It encompasses the study of books, journal articles, datasets, and other scholarly outputs, along with their metadata, such as abstracts, keywords, and citations. The principles of bibliometrics are grounded in the assumption that the scholarly output of a field is reflected in its published literature, allowing researchers to track the spread of topics across the literature and to measure the impact of journal articles, for instance, through their download rates or citation counts (Ninkov et al., 2022). CiteSpace, a bibliometric tool, was developed by Chaomei Chen, a professor of computing and information science at Drexel University. It represents an advanced approach for visualizing and analyzing the evolution of knowledge domains. Rooted in the principles of progressive knowledge domain visualization, CiteSpace employs advanced methodologies, including time slicing, cocitation analysis, and network pruning, to identify pivotal intellectual milestones and temporal patterns within the scientific literature (Chen, 2004). CiteSpace serves as an indispensable instrument for biomedical researchers, facilitating the visual tracking and analytical assessment of evolving research paradigms.

In recent years, CiteSpace has been widely utilized to exploit knowledge domains and emerging trends in HIV-related studies, such as HIV-*Mycobacterium tuberculosis* co-infection (Liu et al., 2023), the effects of exercise on HIV-infected persons (Kose et al., 2021), and pre-exposure prophylaxis (Chen and Lai, 2023). In contrast to conventional literature reviews, CiteSpace-based bibliometric research facilitates visual and quantitative analysis of core research entities, international collaborative networks, overall intellectual architecture, evolutionary trajectories, and research hotspot dynamics within a given research domain. Despite extensive research on the interplay between HIV and malaria, no bibliometric analyses have been conducted to unravel the intricate relationship between HIV infection and malaria systematically. This notable research gap represents a significant limitation, as elucidating the patterns of co-occurrence and potential synergistic interactions between these diseases holds considerable promise for advancing the development of more effective therapeutic approaches and targeted public health interventions. Our study comprehensively analyzes the research landscape on HIV-malaria interactions, emphasizing key theoretical frameworks and critical research domains. By integrating foundational and clinical studies, we utilize CiteSpace to map and visualize the intellectual structure of the field. This method identifies persistent challenges and highlights emerging trends, offering a nuanced and forward-looking perspective on the evolution of HIV and malaria research.

## 2 Methods

### 2.1 Data sources

**Figure 1:**
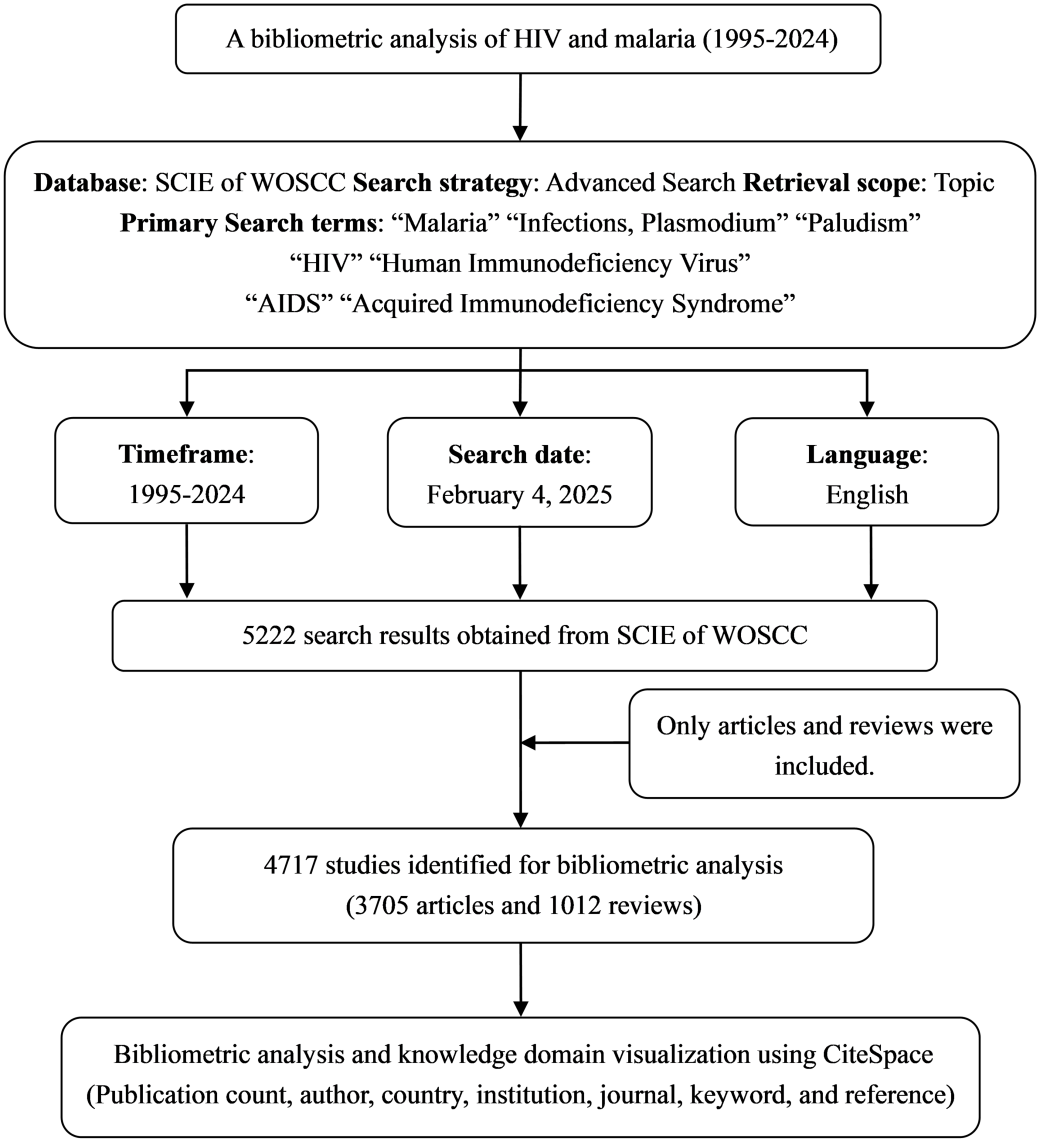
Flow diagram for the collection and analysis of research data pertaining to HIV and malaria interactions.

Web of Science is the foremost resource for citation and bibliometric research, and it is distinguished by its enormous and well-organized universal statistics, surpassing platforms comprising PubMed, Scopus, and prominent Chinese databases, including CNKI and SinoMed. For bibliometric analysis, it is the preferable database. Therefore, the original data was obtained exclusively from the Science Citation Index Expanded (SCIE), a subset of the Web of Science Core Collection (WOSCC). Documents related to HIV and malaria interactions were determined by integrating subject terms and free words. The primary search terms encompassed “Malaria,” “Infections, *Plasmodium*,” “Paludism,” “HIV,” “Human Immunodeficiency Virus,” “AIDS,” and “Acquired Immunodeficiency Syndrome.” The comprehensive search strategies comprising additional search terms are available in Supplementary material S1. Only English-language articles and reviews released between January 1, 1995, and December 31, 2024, were incorporated in this study. The data collection was performed on February 4, 2025. Following the search, 4,717 documents (Supplementary material S2) were gathered, comprising 3,705 articles (78.5%) and 1,012 reviews (21.5%). The chosen papers were downloaded and exported in the form of plain text files (txt) featuring “Full Record and Cited References,” subsequently saved in files named “Download_***txt.” Figure 1 illustrates the search process and selection standards for the study. It is imperative to highlight that our extensive search approach is vital for achieving thorough coverage. Clustering and trend analyses can reveal insights that may be difficult to discover through human observation by automatically filtering out less pertinent or irrelevant documentation.

### 2.2 Bibliometric and visualized analysis

CiteSpace software (version 6.4. R1 Advanced), a user-friendly bibliometric investigation and knowledge graph-creating instrument (Chen, 2004), was employed to perform an analysis of information and visual representation in the research, picturing the multifaceted and fascinating scientific landscapes surrounding HIV and malaria interactions. The relevant parameters for CiteSpace were established as follows: (1) a time slice width of 1 year was established, spanning from 1995 to 2024; (2) a *k*-value of 25 was designated for the g-index; (3) the Top N threshold was determined to be Top 50; (4) network pruning was accomplished utilizing the minimum spanning tree (MST) algorithm; (5) nodes featuring purple outlines exhibit high betweenness centrality values (no less than 0.1). The graphic representation of various dimensions was meticulously analyzed, leveraging cooccurrence mining, dual map overlay, cluster analysis, and burst examination within CiteSpace. In the graph, the color shift from a cool hue (blue) to a warm hue (pink) signifies a temporal progression from the distant past (1995) to the near present (2024).

## 3 Results

### 3.1 Analysis of publication volume

**Figure 2:**
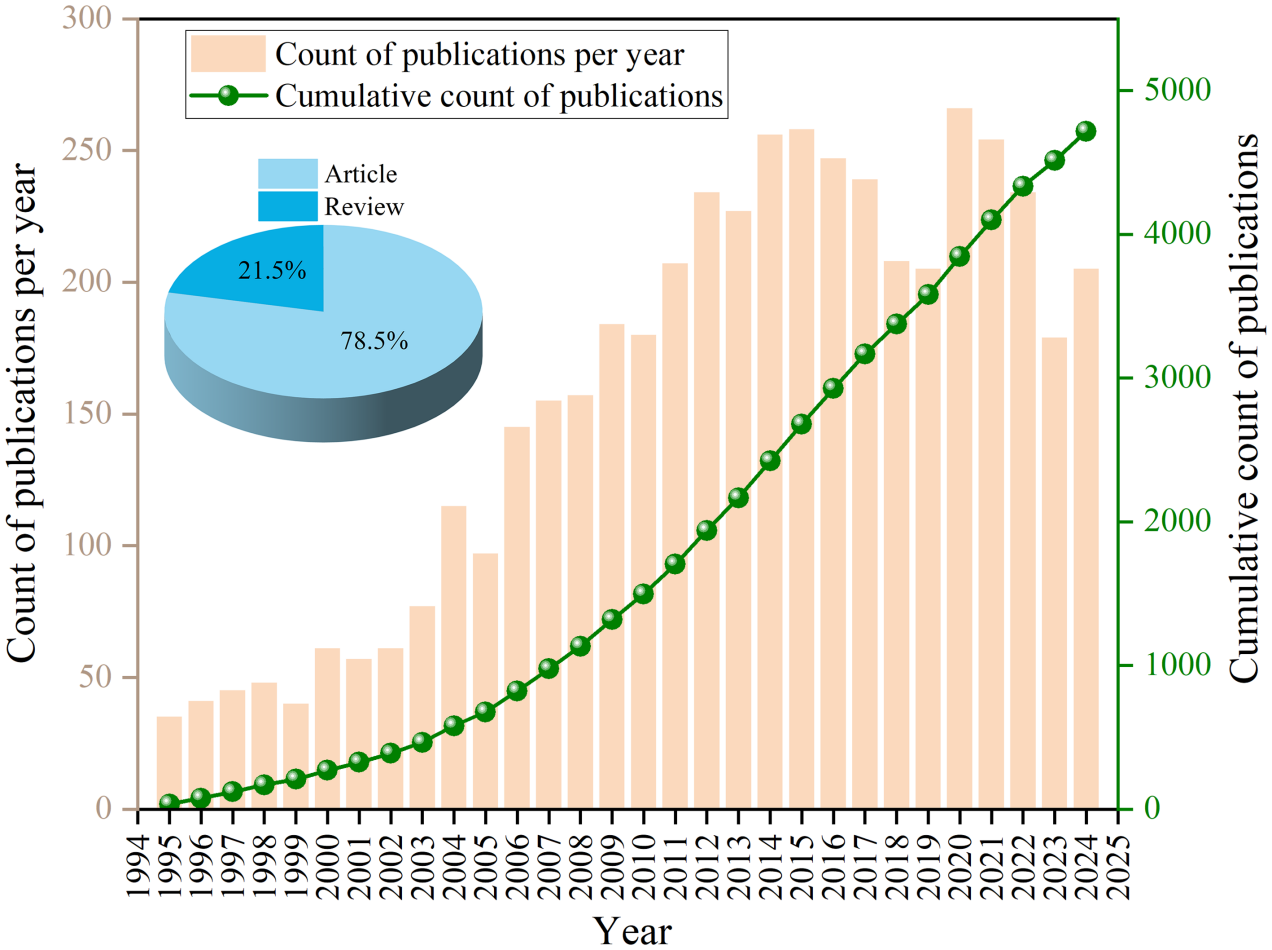
Column diagram and line chart displaying the publication numbers associated with HIV and malaria interactions from 1995 to 2024.

The variations in the number of scholarly publications published each year provide a quantitative depiction of the scholarly endeavors and theoretical development within a specific domain, promoting the review of its historical context and the prediction of forthcoming patterns. Figure 2 illustrates an examination of articles concerning the interactions between HIV and malaria in the WOSCC, revealing a general trend of fluctuating yearly publication volume increases. There are two primary stages in the evolution of publications. In the initial phase (1995–2005), the annual publication count was relatively low, culminating in a zenith of 115 articles in 2004. Throughout the subsequent phase (2006–2024), the research advanced to a phase of growth and stabilization, exhibiting an upward trajectory. The yearly volume of publications at this period was comparatively high, reaching a peak of 266 articles in 2020 and a nadir of 145 in 2006. The peak in the number of articles published in 2020 may be closely associated with COVID-19. These results illuminated the historical development of academic interest and research output in HIV and malaria interactions, suggesting prospects to conduct further exploration.

### 3.2 Analysis of countries and institutions

**Figure 3:**
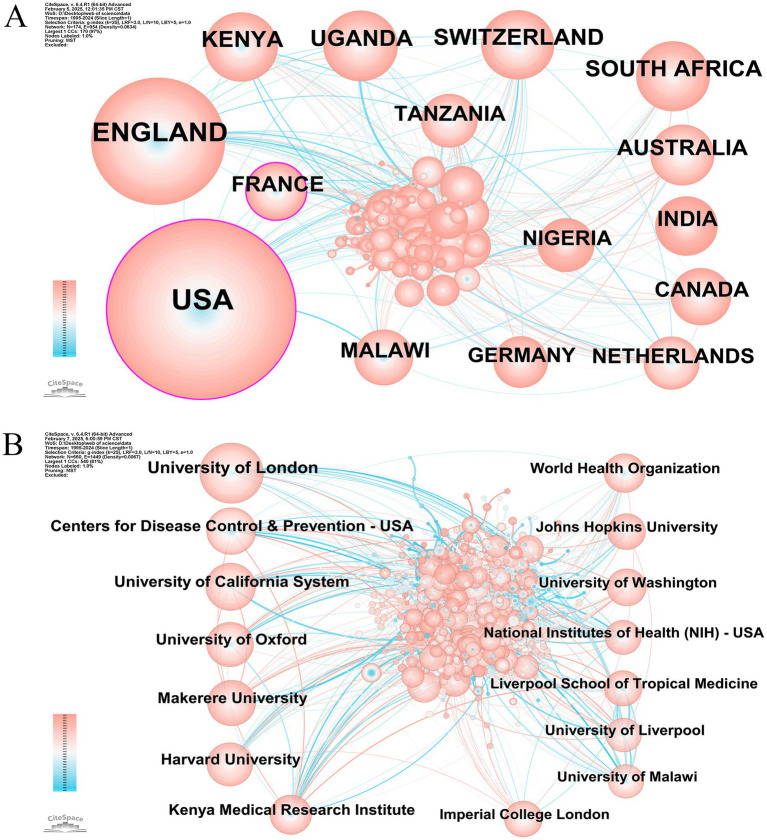
**(A)** International collaborative network of research concerning HIV and malaria interactions. Node = 174, Edge = 954, Density = 0.0634. **(B)** Cooperative network of institutions dedicated to research on HIV and malaria interactions. Node = 660, Edge = 1,449, Density = 0.0067. Original versions of **(A,B)** are included in the Supplementary material.

Transnational and cross-institutional cooperation in scientific research has significantly benefited the global research community, underscoring the collaborative relationships between various countries and research institutions. As illustrated in Figures 3A,B, we utilized CiteSpace to graphically depict the prominent nations and organizations involved in publications on HIV and malaria interactions in the WOSCC in order to gage the degree of their cooperation. The top 15 countries and institutions are presented in Table 1. The United States (1,973; 41.8%) produced the largest number of publications, preceded by England (1,040; 22.0%), Kenya (362, 7.7%), and Uganda (360, 7.6%). In terms of research institutions, the University of London (407, 8.6%) ranked first in total publications, with the Centers for Disease Control and Prevention-United States (267, 5.7%) and the University of California System (255, 5.4%) coming in second and third, respectively. Nodes featured in the national and institutional collaborative network diagrams signify countries or institutions, with their collaboration represented by lines connecting the nodes. Nodes of greater size indicate a higher volume of released papers, whereas lines with more thickness denote a closer degree of cooperation. Figure 3A displays a collaborative network of 174 country-level nodes interconnected through 954 edges, yielding a network density of 0.0634. This comparatively higher density value suggests strong international cooperation patterns, particularly exemplified by the United States. Conversely, Figure 3B reveals a more scattered institutional network configuration consisting of 660 nodes with only 1,449 interconnections, resulting in a significantly lower network density of 0.0067. This sparse network pattern indicates less cooperative engagement between institutions. In particular, the United States and France are depicted as nodes marked with purple outer circles, reflecting their exceptional centrality. This discovery indicates that these nations play a crucial role in the research on HIV and malaria interactions. Nevertheless, the absence of nodes with a significant centrality in Figure 3B underscores the necessity of boosted collaboration and interaction among institutions to produce more significant research findings.

**Table 1:** Top 15 productive nations and institutions in terms of research on HIV and malaria interactions.

Rank	Country	Count	Institution	Count
1	United States	1,973	University of London	407
2	England	1,040	Centers for Disease Control and Prevention-USA	267
3	Kenya	362	University of California System	255
4	Uganda	360	University of Oxford	242
5	Switzerland	355	Makerere University	220
6	South Africa	350	Harvard University	220
7	Australia	274	Kenya Medical Research Institute	197
8	India	256	World Health Organization	182
9	France	248	Johns Hopkins University	173
10	Canada	243	University of Washington	167
11	Malawi	242	National Institutes of Health (NIH) – United States	165
12	Tanzania	222	Liverpool School of Tropical Medicine	163
13	Nigeria	218	University of Liverpool	158
14	Netherlands	216	University of Malawi	156
15	Germany	193	Imperial College London	143

**Figure 4:**
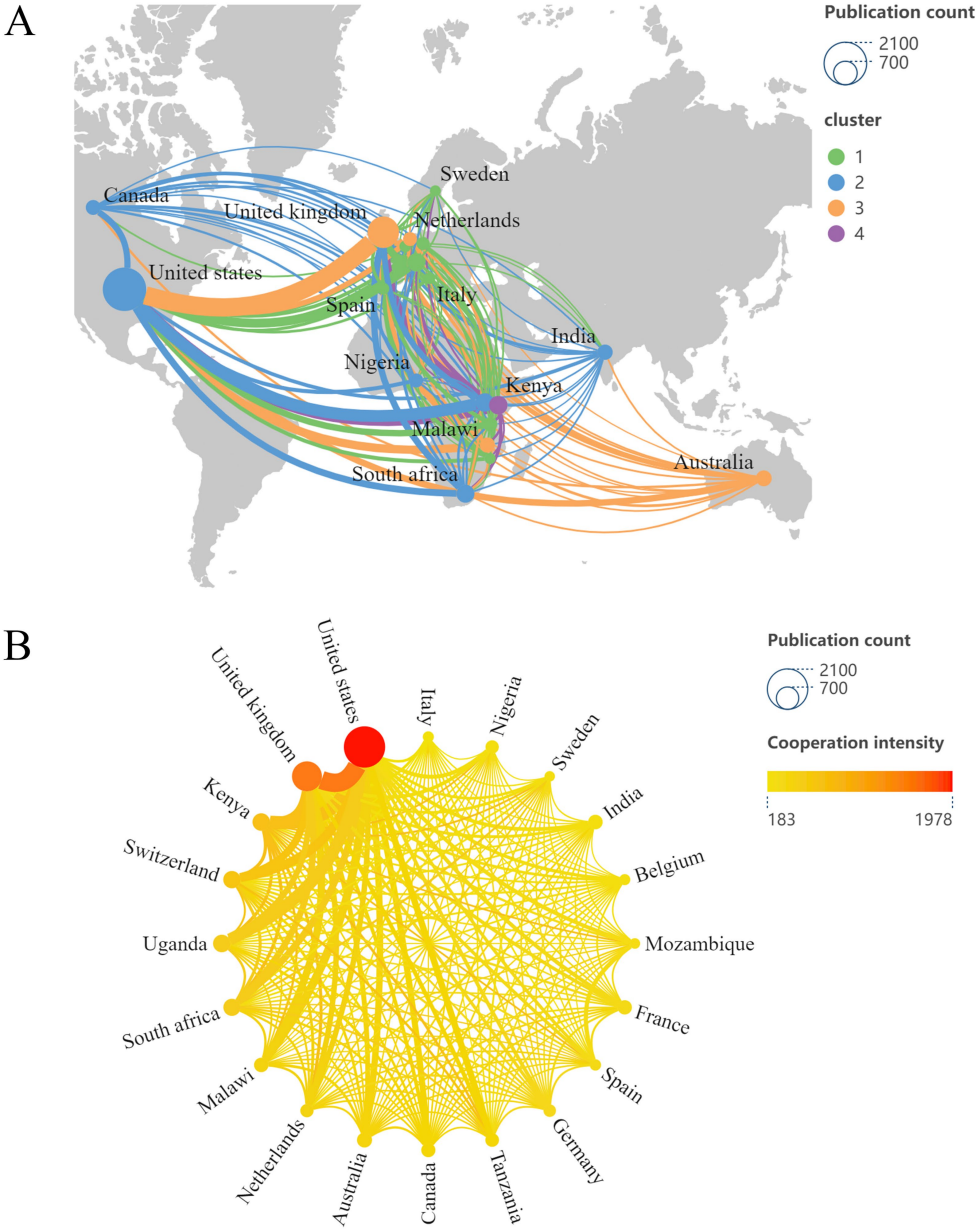
**(A)** Geographical distribution of the impact of HIV and malaria interaction research created with Scimago Graphica and VOSviewer. **(B)** The cooperative network diagram of the leading 20 countries was created with Scimago Graphica and VOSviewer.

Furthermore, we leveraged Scimago Graphica and VOSviewer to generate a geomap (Figure 4A) to efficiently illustrate the geographically distributed characteristics of the research impact. In addition, Figure 4B presents a cooperative network diagram of the leading 20 countries, with the darker and redder nodes representing the more collaborative papers with other countries. In both figures, the dimensions of the nodes represent the quantity of publications produced by each nation, while the thickness of the connections between the two countries is proportional to the robustness of their collaboration. As evidenced by Figures 4A,B, the network of international collaborative efforts is multifaceted and extensive, with the principal hubs for partnership located in America, Europe, and Africa. Notably, the United States possessed the most significant quantity of cooperative publications alongside other nations. The United States and England demonstrated the most intense collaboration, with Kenya and the United States closely following.

### 3.3 Examination of author contributions and cocited author network

**Figure 5:**
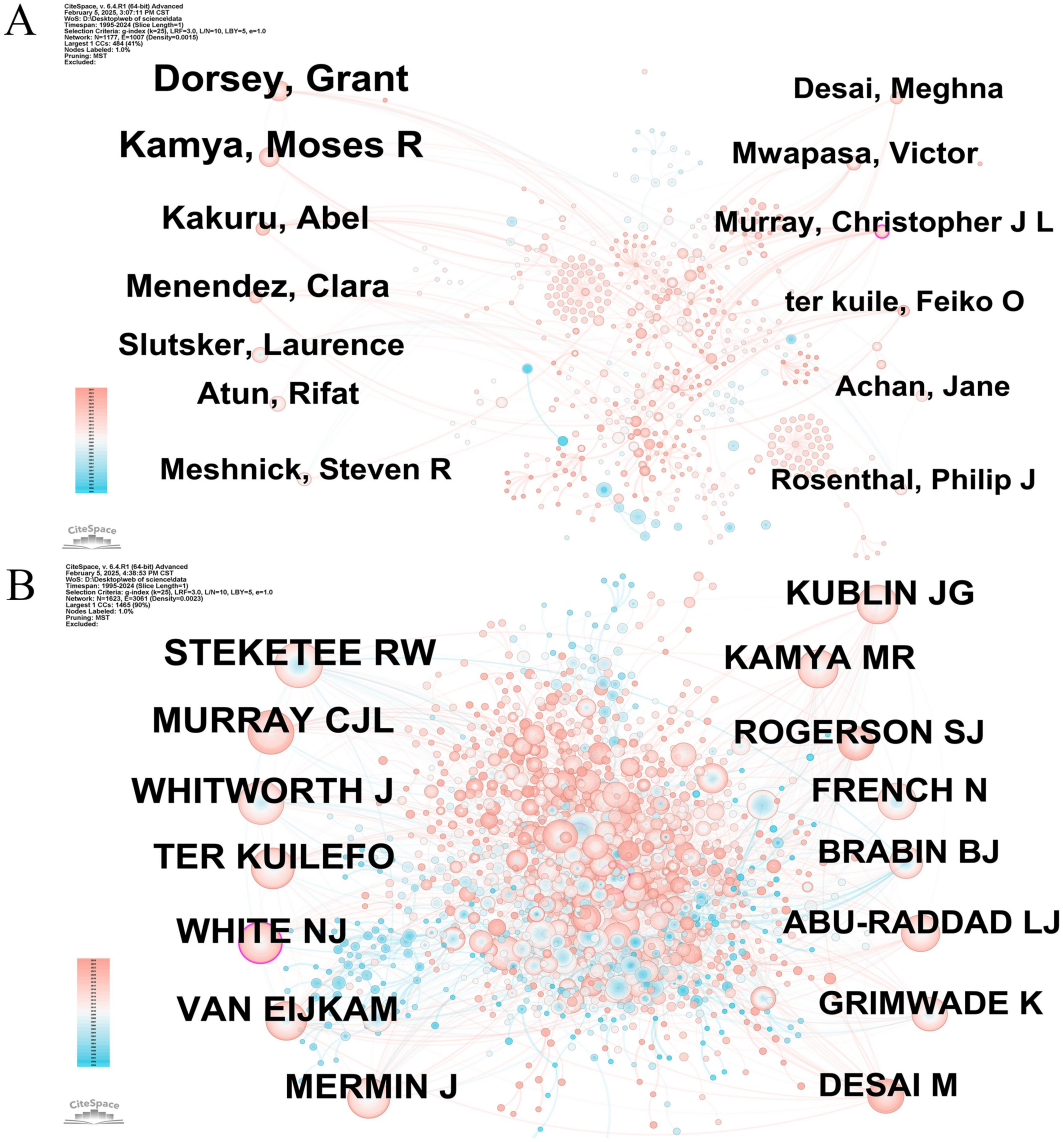
**(A)** Graph depicting authors engaged in studies concerning HIV and malaria interactions. Node = 1,177, Edge = 1,007, Density = 0.0015. **(B)** Graphical illustration of cocited authors involved in HIV and malaria interaction research. Node = 1,623, Edge = 3,061, Density = 0.0023. Original versions of **(A,B)** are included in the Supplementary material.

In order to identify cooperation patterns and determine prominent contributors exhibiting significant impact in HIV-malaria interaction studies, we employed CiteSpace to create an authorship network visualization diagram. Nodes correspond to authors while connecting edges showcase collaborative associations between researchers. Figure 5A exhibits a loose collaborative structure characterized by 1,177 nodes and 1,007 links, yielding an exceptionally sparse network configuration with density metrics measuring 0.0015. This quantitative pattern suggests limited inter-researcher connectivity within the current collaborative network. Cocited authors represent two or more collaborators demonstrating simultaneous citation patterns by one or more academic works, forming a cocited association. This concept is exemplified by the network structural framework depicted in Figure 5B, and the cocited scholars created a relatively strong cocitation connection (1,623 nodes, 3,061 linkages, and a graph density of 0.0023). The 15 foremost scholars are ranked in Table 2 based on their publication counts and cocitation metrics. Grant Dorsey significantly contributed to this field, producing the highest quantity of papers (52 documents). The research conducted by Dorsey and collaborators primarily concentrated on the prevention of malaria in HIV-exposed/infected children and pregnant women in Uganda. For instance, the treatment of uncomplicated malaria with artemisinin-based combination therapies, including artemether-lumefantrine (AL) or dihydroartemisinin-piperaquine (DHA-PPQ), demonstrated efficacy and safety in HIV-infected children undergoing antiretroviral therapy (ART) (Kakuru et al., 2014). In addition, the influence of authors increases proportionally with the number of citations their works obtain. Richard W. Steketee garnered a remarkable 407 citations, reflecting his substantial academic accomplishments and the superiority of his published papers. Steketee and colleagues were primarily dedicated to revealing the interactions between malaria and HIV in maternal and child health. Their research, carried out in Kisumu, Kenya, in 2003, revealed that HIV heightened the risk of malaria during pregnancy (van Eijk et al., 2003) and that women co-infected with HIV and malaria faced an elevated risk of experiencing adverse birth outcomes (Ayisi et al., 2003).

**Table 2:** Top 15 authors and cocited authors in the field of HIV and malaria interactions.

Rank	Author	Count	Cited author	Count
1	Grant Dorsey	52	Richard W. Steketee	237
2	Moses R. Kamya	49	Christopher J. L. Murray	209
3	Abel Kakuru	29	Jimmy Whitworth	201
4	Clara Menéndez	28	Feiko O. Ter Kuile	191
5	Laurence Slutsker	24	Nicholas J. White	175
6	Rifat Atun	24	Anne Maria van Eijk	173
7	Richard W. Steketee	23	Jonathan Mermin	169
8	Bernard L. Nahlen	23	James G. Kublin	155
9	Victor Mwapasa	22	Moses R. Kamya	148
10	Steven R. Meshnick	21	Stephen J. Rogerson	140
11	Meghna Desai	19	Neil French	136
12	Christopher J. L. Murray	19	Bernard J. Brabin	126
13	Feiko O. Ter Kuile	18	Laith J. Abu-Raddad	126
14	Jane Achan	18	Kate Grimwade	126
15	Philip J. Rosenthal	18	Meghna Desai	125

It should be highlighted that Christopher J. L. Murray and Nicholas J. White were circled in purple in Figures 5A,B, respectively, reflecting high centrality values and underscoring the particular significance of the two scholars in the cooccurrence network. Murray and coworkers were principally committed to systematically quantifying the population-level health threats posed by HIV and malaria through a global burden of disease study (Murray et al., 2014) and evaluating the cost-effectiveness of interventions by meta-regression analysis (Silke et al., 2024). These studies provided scientific evidence to advance research on interactions between HIV and malaria and facilitate the optimization of global public health policies. White and colleagues established that severe malaria in HIV-coinfected patients exhibited elevated parasitic loads, increased complications and comorbidities, and a heightened case fatality rate, underscoring the significance of early HIV co-infection detection in the clinical management of severe malarial infections (Hendriksen et al., 2012). Additionally, he and coworkers conducted research on the drug–drug interactions between antiretroviral and antimalarial medications. The findings suggested that the co-administration of AL with nevirapine, efavirenz, and ritonavir/lopinavir manifested significant drug interactions. Due to the easily saturable absorption of lumefantrine, the dosage modifications anticipated to be essential need to be prospectively assessed in individuals dually infected with malaria and HIV (Hoglund et al., 2015).

### 3.4 Source journal and cocited journal assessment

The evaluation of research quality is extremely dependent on the ranking levels of scholarly journals. Statistical analysis identified the top 15 source journals contributing to the 4,717 papers, ordered by publication count, with the results displayed in Table 3. *PLOS One* (220 papers) emerged as the predominant contributor among the leading 15 journals exhibiting the highest release volume, with *Malaria Journal* (159 papers), *American Journal of Tropical Medicine* and *Hygiene* (146 papers), and *Journal of Infectious Diseases* (88 papers) comprising subsequent rankings. The *Lancet*, possessing an impact factor (IF) of 98.4, ranked highest among the leading 15 journals, succeeded by the *Bulletin of the World Health Organization* with an IF of 8.4. According to the 2023 Journal Citation Reports (JCR), most of the foremost 15 high-yield journals are classified in Q1 and Q2, signifying exceptional academic standards.

**Table 3:** The top 15 source journals and cocited journals that are relevant to HIV and malaria interactions.

Rank	Source journal	Publication count	IF (2023)	JCR (2023)	Cocited journal	Cocitation frequency	IF (2023)	JCR (2023)
1	PLOS One	220	2.9	Q1	Lancet	2,860	98.4	Q1
2	Malaria Journal	159	2.4	Q2/Q3	PLOS One	1856	2.9	Q1
3	American Journal of Tropical Medicine and Hygiene	146	1.9	Q2/Q3	Journal of Infectious Diseases	1849	5.0	Q1/Q2
4	Journal of Infectious Diseases	88	5.0	Q1/Q2	American Journal of Tropical Medicine and Hygiene	1735	1.9	Q2/Q3
5	Lancet	76	98.4	Q1	New England Journal of Medicine	1,615	96.3	Q1
6	Clinical Infectious Diseases	69	8.2	Q1/Q1	Clinical Infectious Diseases	1,457	8.2	Q1/Q1
7	AIDS	67	3.4	Q3/Q2/Q3	AIDS	1,431	3.4	Q3/Q2/Q3
8	Vaccine	65	4.5	Q2/Q2	Science	1,233	44.8	Q1
9	JAIDS Journal of Acquired Immune Deficiency Syndromes	60	2.9	Q3/Q2	Malaria Journal	1,200	2.4	Q2/Q3
10	Tropical Medicine and International Health	55	2.6	Q2/Q2	Nature	1,186	50.5	Q1
11	PLOS Neglected Tropical Diseases	54	3.4	Q1/Q1	Proceedings of the National Academy of Sciences of the United States of America	1,150	9.4	Q1
12	BMC Public Health	49	3.5	Q1	Bulletin of the World Health Organization	1,140	8.4	Q1
13	BMC Infectious Diseases	46	3.4	Q2	Tropical Medicine and International Health	1,133	2.6	Q2/Q2
14	Bulletin of the World Health Organization	46	8.4	Q1	Transactions of the Royal Society of Tropical Medicine and Hygiene	1,101	1.9	Q3/Q2
15	Antimicrobial Agents and Chemotherapy	44	4.1	Q2/Q1	PLOS Medicine	1,047	10.5	Q1

Cocited journals denote multiple journals simultaneously cited, emphasizing their importance and impact within a specific domain. A journal’s impact is determined by the quantity of co-citations. The scientific community considers journals exhibiting substantial cocitation amounts to be mainstream. Among the cocited journals, 9 of them obtained cocitations more than 1,200 times. As displayed in Table 3, *the Lancet* experiences the most cocitations, with a frequency of 2,860, followed by *PLOS One* with 1,856 cocitations and the *Journal of Infectious Diseases* with 1,849 cocitations. Within the foremost 15 journals ranked by cocitation frequency, the journal with the highest IF was the *Lancet* (98.4), with the *New England Journal of Medicine* following at an IF of 96.3. In addition, the outstanding intellectual foundations of HIV and malaria are illustrated by the classification of 10 of the forefront 15 cocited journals as Q1 by JCR.

**Figure 6:**
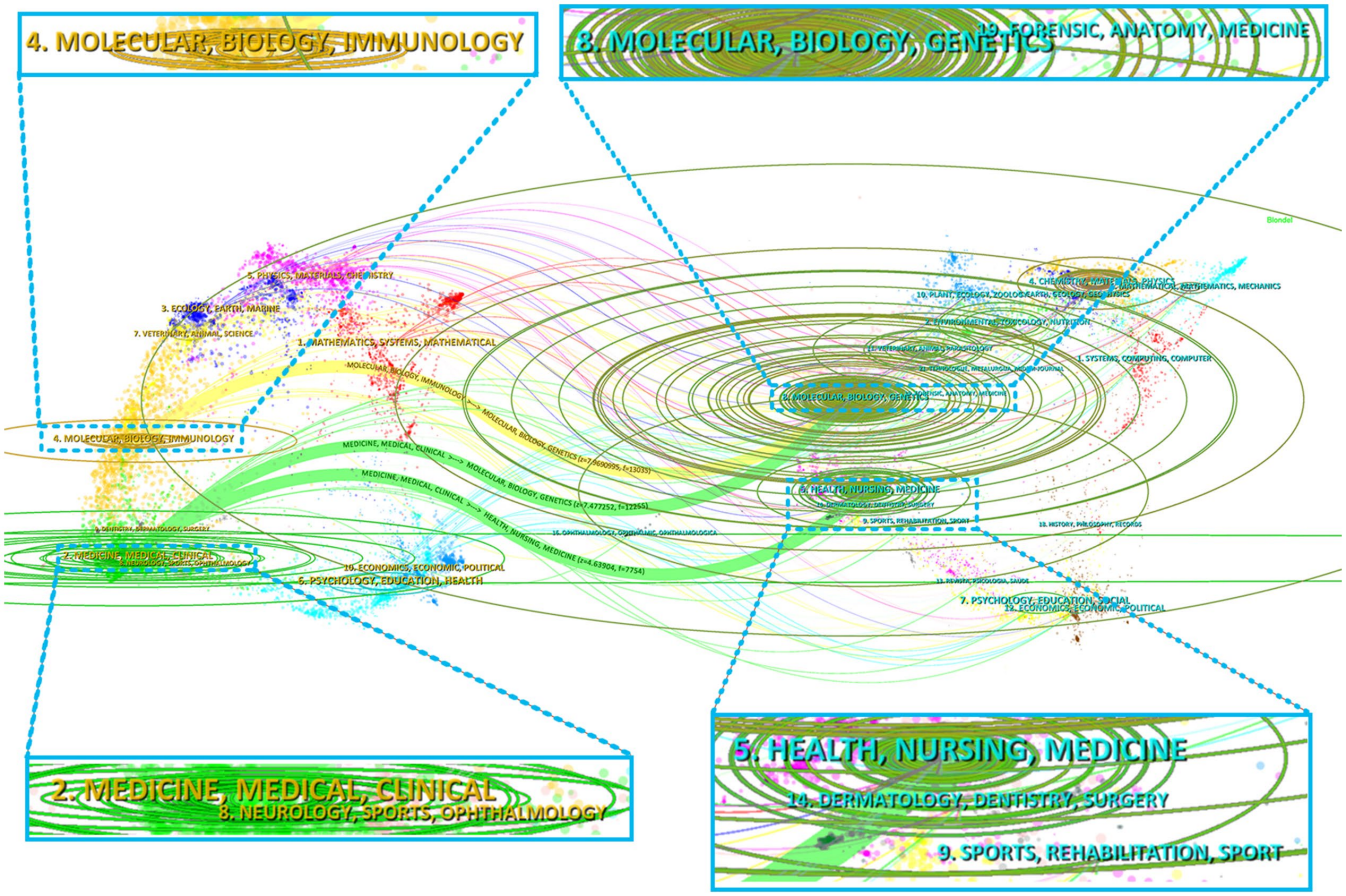
Dual-map overlay of journals pertinent to HIV and malaria interaction research.

The implementation of dual-map overlaying brilliantly portrays the referencing relationships between citing and cocited journals, highlighting journals from different academic disciplines (Figure 6). The right side of the graph displays the collections of cocited journals, while the left side showcases the assemblages of citing journals. The citation connections are presented in Figure 6 by utilizing colored curves. According to the yellow citation track, studies published in Molecular/Biology/Immunology journals frequently referenced research from Molecular/Biology/Genetics journals (frequency = 13,035). As depicted by the green streams, papers released in Molecular/Biology/Genetics journals (frequency = 12,255) and Health/Nursing/Medicine journals (frequency = 7,754) were frequently cited in research from Medicine/Medical/Clinical journals. Table 4 concisely delineates these citation trajectories. The findings revealed a multidisciplinary commitment and the pivotal contributions of these disciplines to investigations into HIV and malaria interactions, involving basic, clinic, and nursing.

**Table 4:** The citation relationships between citing and cocited journals in HIV-malaria interaction research.

Citing domains	Cited domains	Frequency
Molecular/Biology/Immunology	Molecular/Biology/Genetics	13,035
Medicine/Medical/Clinical	Molecular/Biology/Genetics	12,255
Medicine/Medical/Clinical	Health/Nursing/Medicine	7,754

### 3.5 Exploration of keyword patterns

#### 3.5.1 Cooccurrence mapping of keywords

**Figure 7:**
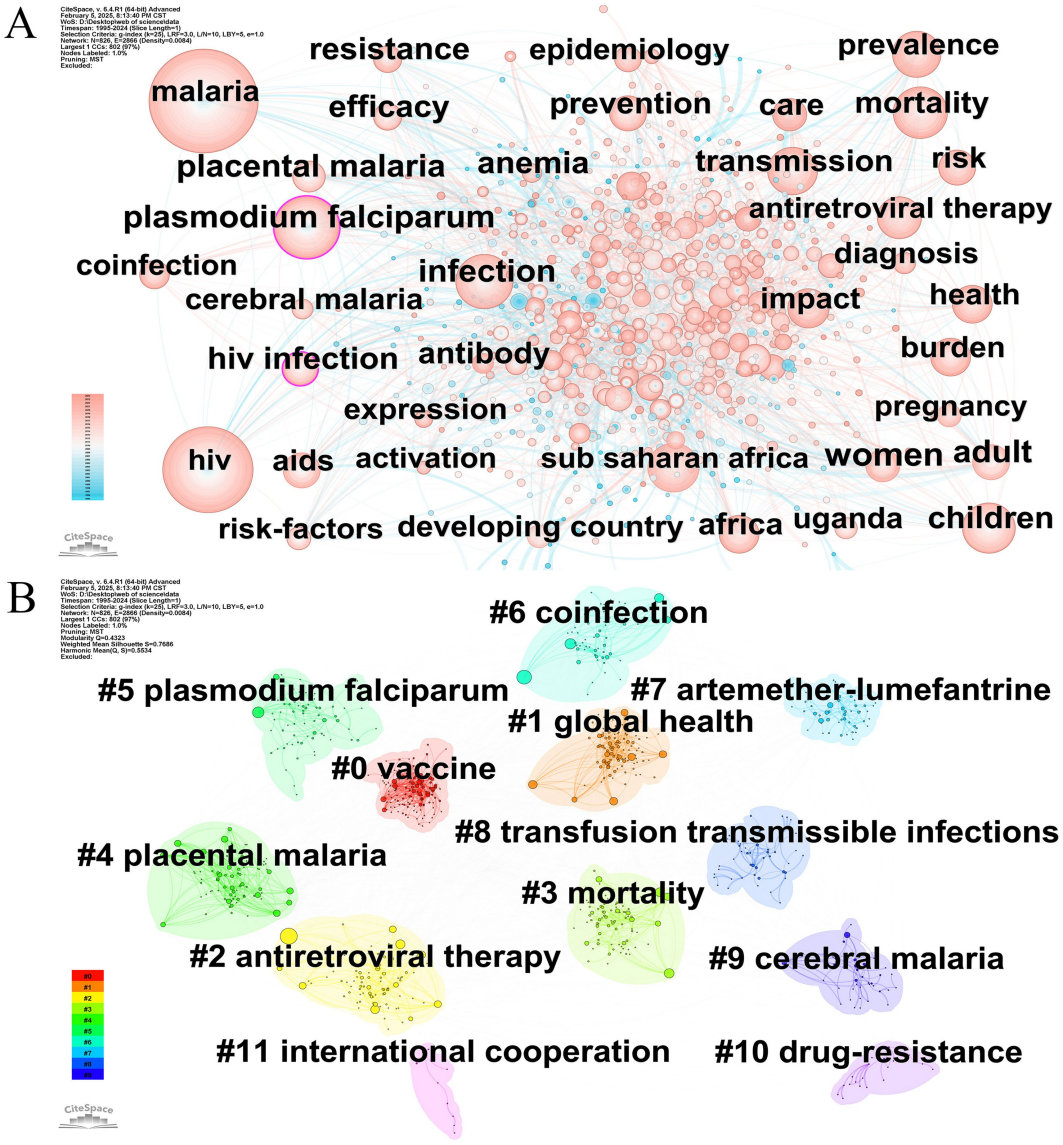
**(A)** Keyword cooccurrence analysis. Node = 826, Edge = 2,866, Density = 0.0084. **(B)** Keyword cluster analysis. Modularity value Q = 0.4323. Mean silhouette value S = 0.7686. Original versions of **(A,B)** are included in the Supplementary material.

An academic paper’s keywords present a succinct overview of the topic of the study. Keyword analysis promotes identifying dominant research interests. The 15 primary keywords displaying high frequency and centrality are outlined in Table 5. Figure 7A showcases a keyword cooccurrence map, encompassing 826 nodes and 2,866 relationships. Among them, it was observed that five keywords occurred more than 300 times, and 11 keywords appeared more than 200 times. The most frequently occurring keywords comprised “malaria,” “HIV,” “*Plasmodium falciparum*,” “infection,” “children,” “mortality,” and “prevalence,” with occurrences of 913, 697, 407, 387, 309, 281, and 276, respectively. Significantly, the purple boundary keywords “*Plasmodium falciparum*” and “HIV infection” exhibited the highest centrality values of 0.13 and 0.10, respectively. The aforementioned high-frequency keywords and cooccurrence network indicate that studies on pediatric populations and epidemiological explorations are predominant topics in the research domain of HIV and malaria interactions.

**Table 5:** Top 15 keywords ranked according to frequency and centrality.

Rank	Keyword	Frequency	Keyword	Centrality
1	Malaria	913	Plasmodium falciparum	0.13
2	HIV	697	HIV infection	0.1
3	Plasmodium falciparum	407	Virus	0.07
4	Infection	387	Aids	0.06
5	Children	309	Antibody	0.06
6	Mortality	281	Cerebral malaria	0.06
7	Prevalence	276	Efficacy	0.06
8	Transmission	265	Infection	0.06
9	sub Saharan Africa	246	Placental malaria	0.06
10	Aids	226	Resistance	0.06
11	Antiretroviral therapy	211	Adult	0.05
12	Africa	196	Antigen	0.05
13	Impact	186	Care	0.05
14	Health	174	Children	0.05
15	HIV infection	168	Expression	0.05

#### 3.5.2 Keyword cluster analysis

After examining keyword cooccurrence, we first grouped the keywords, subsequently exploiting the log-likelihood ratio method to determine nominal terms functioning as cluster markers, thereby accurately delineating the dominant themes and research horizons concerning HIV and malaria interactions. Two indicators, mean silhouette value S and modularity value Q, were employed to evaluate the quality of clustering. Clusters generally display a significant clustering characteristic when the Q value exceeds 0.3. A greater Q value indicates a superior clustering performance. Moreover, clustering is typically considered highly trustworthy if the S value surpasses 0.7. The clusters were appropriately classified into a reasonable framework, as evidenced by the Q value of 0.4323 and the S value of 0.7686 in Figure 7B. The yielded clusters can be primarily divided into four groups. The first category concentrates on the prevention and treatment approaches of HIV and malaria co-infection, comprising #0 vaccine, #2 antiretroviral therapy, and #7 artemether-lumefantrine. The second category is dedicated to the clinical complexity and vertical/transfusion-related transmission mechanisms of HIV and malaria co-infection, which includes #4 placental malaria, #5 *Plasmodium falciparum*, #6 coinfection, #8 transfusion transmissible infections, and #9 cerebral malaria. The disease burden studies and public health research of HIV and malaria co-infection, categorized as the third category, contain #1 global health, #3 mortality, and #11 international cooperation. Cluster #10 drug-resistance is classified under the fourth category. The above findings provide a lucid and intuitive elucidation of the various subtopics underlying the context of HIV and malaria interactions.

#### 3.5.3 Detection of keyword bursting

**Figure 8:**
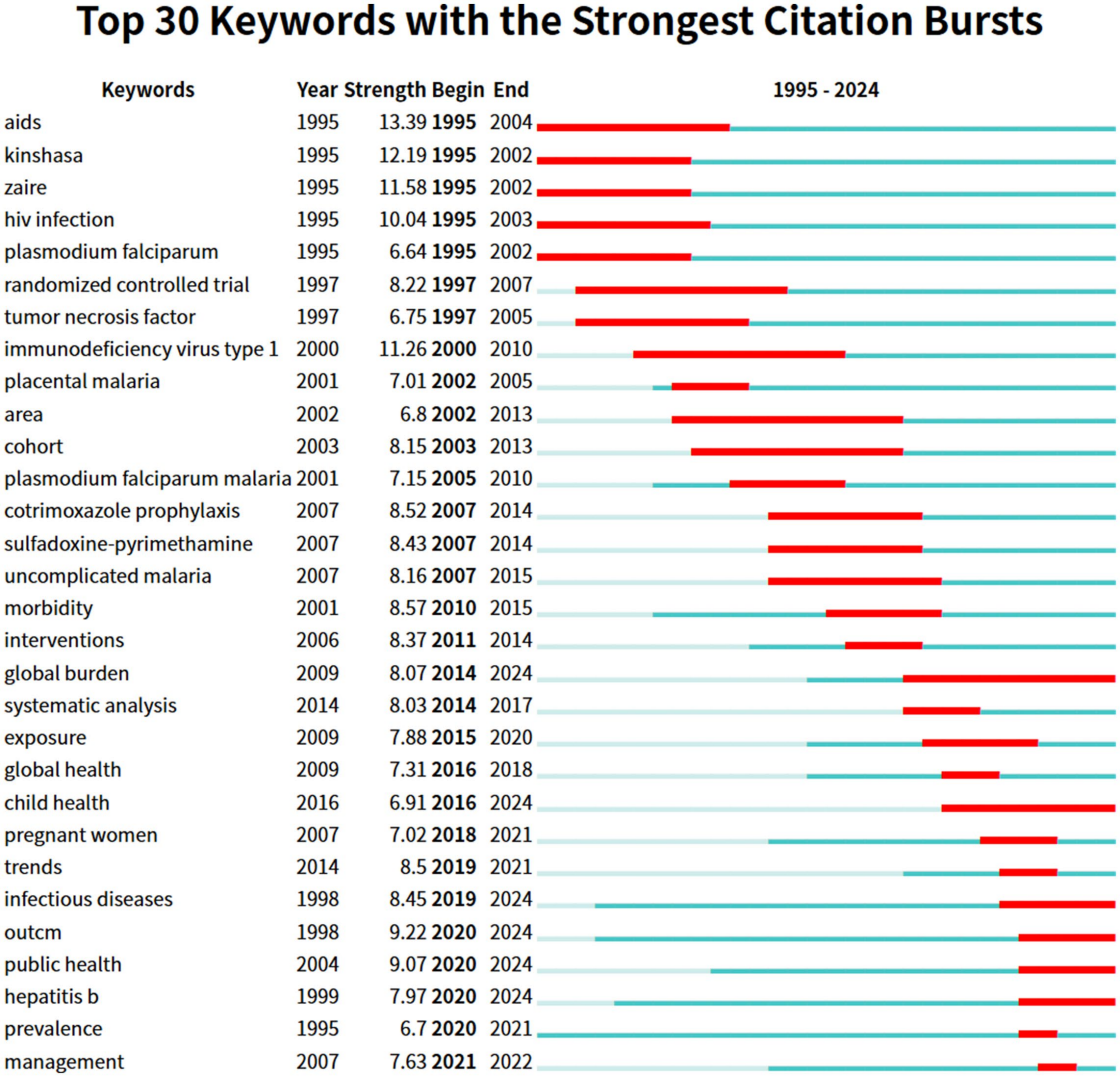
Keyword burst analysis.

Keywords with a substantial citation increase during a brief timeframe are designated as burst keywords. Spotting and analyzing burst keywords can reveal the research forefront within a specific period and aid in identifying emerging academic trends. Figure 8 highlights the top 30 burst keywords with the red line representing the duration of citation bursting and the blue line denoting the time frame. In the initial phases researchers primarily concentrated on exploring the clinical interactions between HIV and malaria and preventative strategies of HIV and malaria co-infection utilizing methodologies such as randomized controlled trials and cohort studies as evidenced by the keywords including disease-related keywords (represented by “AIDS” “HIV infection” “immunodeficiency virus type 1” “*Plasmodium falciparum*” “*Plasmodium falciparum* malaria” “placental malaria” and “uncomplicated malaria”) therapy-related keywords (represented by “cotrimoxazole prophylaxis” and “sulfadoxine-pyrimethamine”) and research design-related keywords (represented by “randomized controlled trial” and “cohort”). A prospective cohort study revealed that placental malaria elevated the risk of mother-to-child HIV transmission (MTCT) independent of maternal HIV viral load highlighting the necessity for improved preventive measures against malaria during pregnancy to reduce the risk of undesirable birth results and MTCT (Brahmbhatt et al., 2008). A randomized controlled trial conducted in Tororo Uganda indicated that artemisinin-based combination treatments specifically AL and DHA-PPQwere well-tolerated and safe for treating uncomplicated malaria in young children who were either HIV-infected or uninfected (Katrak et al., 2009).

Since 2010, there has been a consistent increase in the significance of keywords involving “morbidity,” “global health,” “pregnant women,” “prevalence,” and “management.” This suggests that scholars have gradually shifted their focus to the public health level, investigating the global burden, epidemiological patterns, and effects of HIV and malaria co-infection on particular populations. This phenomenon may be due to the declining global incidence of malaria and HIV, underscoring the imperativeness of updating epidemiological data regarding the disease burden associated with HIV-malaria co-infection. Additionally, it is imperative to prioritize significant keywords that continually emerged until 2024, including “global burden,” “child health,” “infectious diseases,” “outcome,” “public health,” and “Hepatitis B,” since these terms may hint at prospective subjects for subsequent studies. Consequently, in the forthcoming years, epidemiological research and the prevention and management of co-infection with multiple pathogens from a public health standpoint may become the focal point of future research. The sequential appearance of burst keywords reflects the evolutionary pathway and emerging research tendencies on HIV and malaria interactions.

### 3.6 Evaluation of cocited reference patterns

#### 3.6.1 Cooccurrence mapping of cocited references

**Figure 9:**
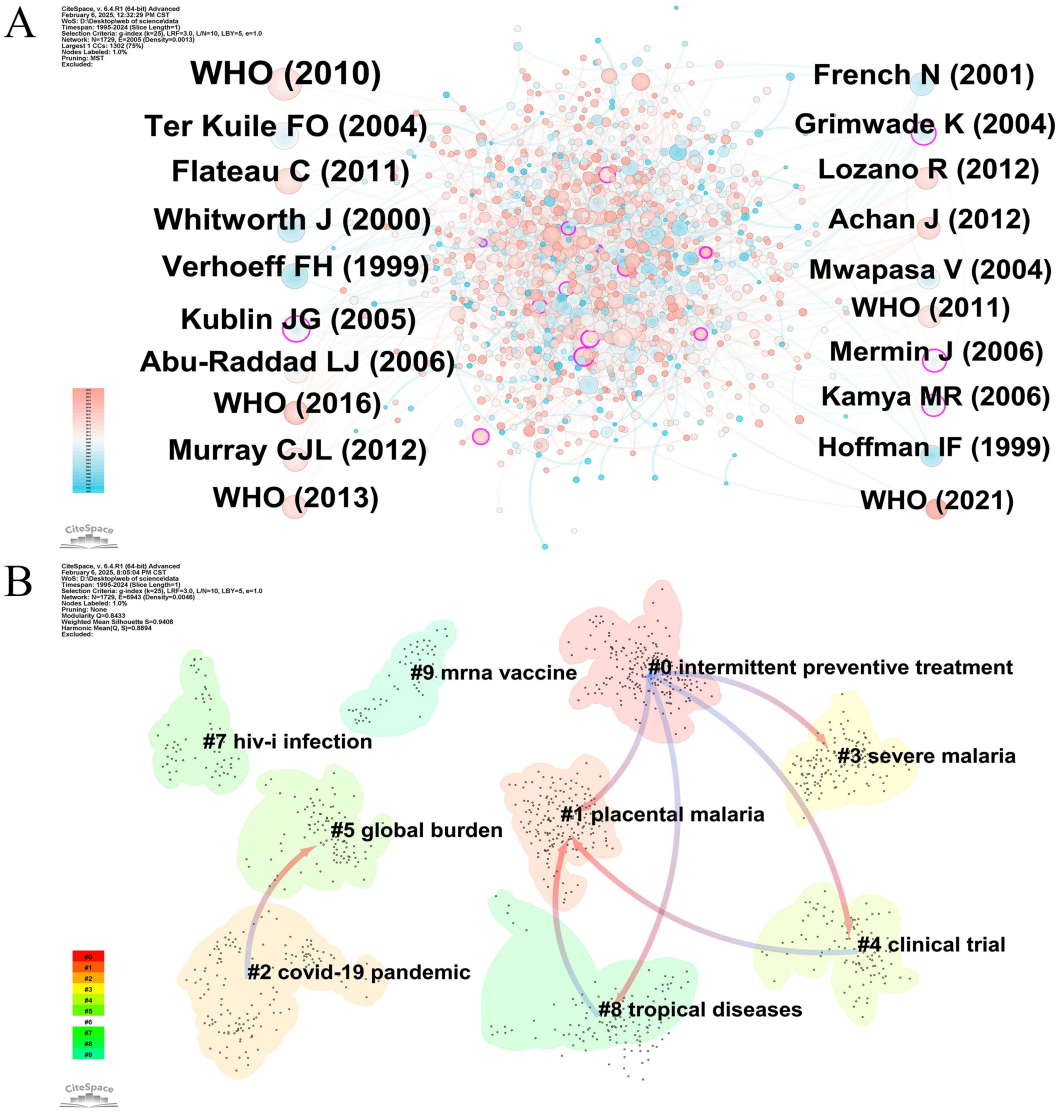
**(A)** The cooccurrence network of cocited references involved in HIV and malaria interaction research. Node = 1,729, Edge = 2,005, Density = 0.0013. **(B)** Cluster dependency map of the cocited references. Modularity value Q = 0.8433. Mean silhouette value S = 0.9408. Original versions of **(A,B)** are included in the Supplementary material.

References that are cited by the researcher concurrently are referred to as cocited references, which are frequently recognized as the foundation of a particular research domain. Figure 9A displays the literature interconnections in the domain of HIV and malaria interactions during the previous three decades through the cooccurrence structure of cocited references. Table 6 lists the 15 most commonly cited references derived from academic journals, with the prior eight receiving greater than 40 citations. Among them, ter Kuile et al. (2004), Flateau et al. (2011), and Whitworth et al. (2000) exhibited cocitation frequencies of 53, 52, and 52, respectively. Notably, within the citation cooccurrence network, nodes featuring significant centrality play a vital intermediate role, displaying substantial influence throughout the network. Table 7 summarizes the 15 most prominent cocited references based on centrality, with Sandison et al. (2011) and Kamya et al. (2007) occupying the top two positions. The aforementioned exceptionally influential articles represent the landmark research of the field.

**Table 6:** Ranking of the top 15 cocited references based on their frequency of citation.

Rank	Cocited reference	Author	Year	Journal	Citation
1	The burden of co-infection with human immunodeficiency virus type 1 and malaria in pregnant women in sub-saharan Africa	Feiko O. ter Kuile	2004	American Journal of Tropical Medicine and Hygiene	53
2	Effect of HIV-1 and increasing immunosuppression on malaria parasitaemia and clinical episodes in adults in rural Uganda: a cohort study	James Whitworth	2000	Lancet	52
3	Consequences of HIV infection on malaria and therapeutic implications: a systematic review	Clara Flateau	2011	Lancet Infectious Diseases	52
4	Increased prevalence of malaria in HIV-infected pregnant women and its implications for malaria control	Francine H. Verhoeff	1999	Tropical Medicine and International Health	47
5	Effect of Plasmodium falciparum malaria on concentration of HIV-1-RNA in the blood of adults in rural Malawi: a prospective cohort study	James G. Kublin	2005	Lancet	44
6	Dual Infection with HIV and Malaria Fuels the Spread of Both Diseases in Sub-Saharan Africa	Laith J. Abu-Raddad	2006	Science	44
7	Global malaria mortality between 1980 and 2010: a systematic analysis	Christopher J. L. Murray	2012	Lancet	42
8	Increasing rates of malarial fever with deteriorating immune status in HIV-1-infected Ugandan adults	Neil French	2001	AIDS	40
9	HIV infection as a cofactor for severe falciparum malaria in adults living in a region of unstable malaria transmission in South Africa	Kate Grimwade	2004	AIDS	38
10	Global and regional mortality from 235 causes of death for 20 age groups in 1990 and 2010: a systematic analysis for the Global Burden of Disease Study 2010	Rafael Lozano	2012	Lancet	38
11	Antiretroviral agents and prevention of malaria in HIV-infected Ugandan children	Jane Achan	2012	New England Journal of Medicine	37
12	The effect of Plasmodium falciparum malaria on peripheral and placental HIV-1 RNA concentrations in pregnant Malawian women	Victor Mwapasa	2004	AIDS	35
13	Effect of co-trimoxazole prophylaxis, antiretroviral therapy, and insecticide-treated bednets on the frequency of malaria in HIV-1-infected adults in Uganda: a prospective cohort study	Jonathan Mermin	2006	Lancet	34
14	The effect of Plasmodium falciparum malaria on HIV-1 RNA blood plasma concentration	Irving F. Hoffman	1999	AIDS	33
15	Effect of HIV-1 infection on antimalarial treatment outcomes in Uganda: a population-based study	Moses R. Kamya	2006	Journal of Infectious Diseases	33

**Table 7:** Top 15 cocited references ranked in accordance with centrality.

Rank	Cocited reference	Author	Year	Journal	Centrality
1	Protective efficacy of co-trimoxazole prophylaxis against malaria in HIV exposed children in rural Uganda: a randomized clinical trial	Taylor G. Sandison	2011	BMJ	0.23
2	Effects of trimethoprim-sulfamethoxazole and insecticide-treated bednets on malaria among HIV-infected Ugandan children	Moses R. Kamya	2007	AIDS	0.21
3	HIV-1/AIDS and maternal and child health in Africa	François Dabis	2002	Lancet	0.18
4	Intrapartum and neonatal single-dose nevirapine compared with zidovudine for prevention of mother-to-child transmission of HIV-1 in Kampala, Uganda: HIVNET 012 randomized trial	Laura A. Guay	1999	Lancet	0.17
5	Malaria	Nicholas J. White	2014	Lancet	0.16
6	Effect of co-trimoxazole prophylaxis, antiretroviral therapy, and insecticide-treated bednets on the frequency of malaria in HIV-1-infected adults in Uganda: a prospective cohort study	Jonathan Mermin	2006	Lancet	0.15
7	The effect of malaria and malaria prevention in pregnancy on offspring birthweight, prematurity, and intrauterine growth retardation in rural Malawi	Richard W. Steketee	1996	American Journal of Tropical Medicine and Hygiene	0.15
8	Rates and risk factors for mortality during the first two years of life in rural Malawi	Peter Bloland	1996	American Journal of Tropical Medicine and Hygiene	0.14
9	Beyond malaria--causes of fever in outpatient Tanzanian children	Valérie D’Acremont	2014	New England Journal of Medicine	0.14
10	Effect of HIV-1 infection on antimalarial treatment outcomes in Uganda: a population-based study	Moses R. Kamya	2006	Journal of Infectious Diseases	0.13
11	Comparability of treatment groups and risk factors for parasitemia at the first antenatal clinic visit in a study of malaria treatment and prevention in pregnancy in rural Malawi	Richard W. Steketee	1996	American Journal of Tropical Medicine and Hygiene	0.13
12	HIV infection as a cofactor for severe falciparum malaria in adults living in a region of unstable malaria transmission in South Africa	Kate Grimwade	2004	AIDS	0.12
13	Effect of Plasmodium falciparum malaria on concentration of HIV-1-RNA in the blood of adults in rural Malawi: a prospective cohort study	James G. Kublin	2005	Lancet	0.11
14	Relation between falciparum malaria and bacteraemia in Kenyan children: a population-based, case–control study and a longitudinal study	J Anthony G. Scott	2011	Lancet	0.10
15	Potential impact of the COVID-19 pandemic on HIV, tuberculosis, and malaria in low-income and middle-income countries: a modeling study	Alexandra B. Hogan	2020	Lancet Global Health	0.09

These seminal studies on HIV and malaria interactions can be categorized into three major themes. The first category of themes is epidemiological studies, focusing on disease burden and disease spread. ter Kuile et al. observed that HIV transformed the conventional gravidity-specific malaria risk profile, switching the burden from predominantly primigravidae and secundigravidae to encompass all pregnant women. In populations exhibiting HIV prevalences of 10 and 40%, the estimated proportionate rise for malaria during pregnancy caused by HIV was 5.5 and 18.8%, respectively (ter Kuile et al., 2004). Abu-Raddad et al. spotted that co-infection with HIV and malaria might promote the geographical spread of malaria in regions with higher HIV prevalence, and temporary and recurring raises in HIV viral loads due to repeated co-infection with malaria might significantly contribute to the dissemination of HIV in sub-Saharan Africa (Abu-Raddad et al., 2006).

The second theme involves the efficacy of clinical management and preventive intervention, including co-trimoxazole prophylaxis, antiretroviral agents, and insecticide-treated bednets (ITNs). Sandison et al. illustrated that co-trimoxazole prophylactic treatments provided moderate protection against malaria in HIV-exposed infants when administered beyond the scope of HIV exposure duration, notwithstanding the high prevalence of *Plasmodium* genotypes linked to antifolate resistance (Sandison et al., 2011). Kamya et al. demonstrated that in a malaria-endemic area with a high proportion of molecular markers for antifolate resistance, the concurrent application of co-trimoxazole preventative drugs and ITNs significantly reduced the malaria incidence among HIV-infected children (Kamya et al., 2007). Mermin et al. confirmed that the integration of co-trimoxazole, ART, and ITNs effectively decreased the incidence of malaria in adults infected with HIV in Uganda (Mermin et al., 2006).

The third theme focuses on biological mechanisms and pathophysiological interactions. Whitworth et al. focused on the effect of HIV-1 on malaria and concluded that HIV-1 infection was linked to a higher incidence of clinical malaria and *falciparum* parasitemia, with this correlation becoming more evident as immunosuppression progressed (Whitworth et al., 2000). The effects of malaria on HIV infection were explored by Hoffman et al. (1999), Mwapasa et al. (2004), and Kublin et al. (2005), and all three studies showed that infection with *P. falciparum* malaria was associated with a rise in HIV-1 viral load. Flateau et al. described the effects of HIV infection on malaria. HIV infection was associated with increased prevalence and severity of clinical malaria and reduced response to antimalarial treatment, depending on age, immunosuppression, and previous malarial immunity. HIV also affected the response to intermittent preventive treatment and pregnancy-related immunity to malaria. ART and co-trimoxazole preventative measures reduced the incidence of clinical malaria (Flateau et al., 2011).

#### 3.6.2 Reference cluster dependency analysis

Based on the cocitation connections, Figure 9B showcases the clusters of cocited references and interdependencies among different clusters. With Q and S metrics reaching 0.8433 and 0.9408, respectively, the results reveal a significant reference clustering outcome and demonstrate an appropriate structural distribution of clusters. The establishment of a particular cluster is contingent upon other clusters, which is referred to as cluster dependency. The dependencies among various clusters were streamlined by selecting the primary 30% of cluster dependency tracks, as portrayed in Figure 9B, enhancing visual representation and understanding of the interconnections across the clusters for scholars. The interdependencies that exist between clusters are represented by the colored curved lines with arrows. For instance, the arrow link from #2 COVID-19 pandemic to #5 global burden implies that the papers from #5 were cited by the papers from #2, reflecting that cluster #5 served as the intellectual basis for cluster #2. Following the outbreak of the COVID-19 pandemic, numerous studies focused on the impact of COVID-19 on HIV and malaria. A modeling analysis indicated that interruptions to HIV and malaria services caused by the COVID-19 pandemic could lead to a significant rise in HIV and malaria deaths in areas with high burdens of these diseases. This study also revealed that sustaining the essential services, particularly ART for HIV and the distribution of long-lasting ITNs and prophylactic treatment for malaria, could dramatically mitigate the overall effects of the COVID-19 pandemic, underscoring the necessity of enhancing the resilience of the health system to withstand disruptive occurrences such as emerging pandemics (Hogan et al., 2020). In conclusion, this interconnectedness highlights the development and evolution of research on HIV and malaria interactions.

#### 3.6.3 Reference burst analysis

**Figure 10:**
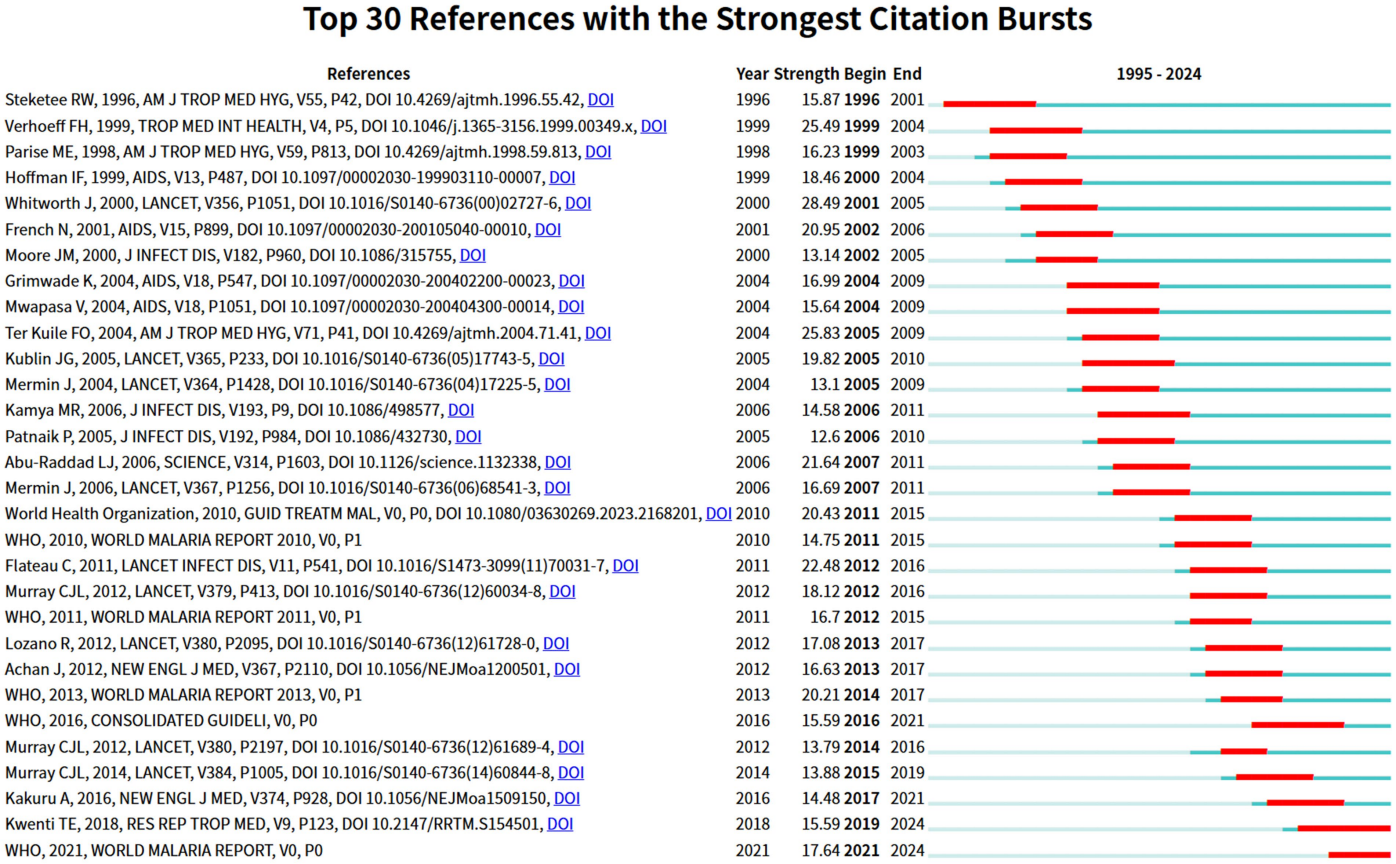
Burst analysis of cocited references related to HIV and malaria interactions.

A burst reference is distinguished by an upsurge in citation frequency that appears during a specific period. Figure 10 displays the 30 most prominent references that exhibit the most notable citation outbursts. Based on this figure, the first research article experiencing a citation surge was produced by Steketee et al., which received considerable attention from 1996 to 2001, showcasing a burst intensity of 15.87. This study investigated the correlation between HIV and *P. falciparum* in pregnant women, confirming that HIV infection reduced the ability of pregnant women to prevent *P. falciparum* parasitemia as well as placental and neonatal infections (Steketee et al., 1996). It should be noted that the paper by Whitworth et al., titled “Effect of HIV-1 and increasing immunosuppression on malaria parasitaemia and clinical episodes in adults in rural Uganda: a cohort study,” exhibited the strongest burst strength value (28.49) from 2001 to 2005. In addition, it is noteworthy that two references are experiencing a citation spike until 2023, indicating that they may continue to explode in future timeframes. The publication entitled “Malaria and HIV coinfection in sub-Saharan Africa: prevalence, impact, and treatment strategies,” authored by Tebit E. Kwenti and published in *Research and Reports in Tropical Medicine*, demonstrated a burst strength of 15.59, with a burst of citations spanning from 2019 to 2024. This review thoroughly summarized the prevalence, impact, and therapeutic approaches of HIV and malaria co-infection in sub-Saharan Africa. The author also identified potential directions for further research, including elucidating the effects of malaria on HIV, clarifying drug–drug interactions in patients concurrently receiving antimalarials and antiretroviral medications, and developing more secure and more economical medications and vaccines for preventing malaria in HIV-infected pregnant women (Kwenti, 2018). Particularly, the scholarly literature derived through cocited reference cooccurrence investigation and burst examination shows a firm consistency, thereby confirming the importance of these selected publications as key articles in this area.

## 4 Discussion

Employing CiteSpace for visualization, the present research examined the distribution of papers associated with HIV and malaria interactions across countries, organizations, contributors, cocited researchers, journals, cocited journals, keywords, and cocited references, meticulously clarifying the current landscape, identifying key hotspots and frontiers, and probing emerging trends in this field. Over the preceding three decades, the field of HIV and malaria interactions has attracted substantial scholarly attention and yielded significant breakthroughs. Through cooperation network analysis, we determined the most prolific nations, organizations, and scholars and constructed a three-tiered collaboration framework encompassing macro-level national coordination, meso-level institutional networks, and micro-level individual researcher engagement. In particular, collaborative relationships at both institutional and investigator levels should be systematically strengthened. Regarding journals and cocited journals, it is indisputable that most of them possess a significant worldwide influence. Based on the examination of keywords and cocited references, the research hotspots, frontiers, and trends in the HIV and malaria interaction field are systematically summarized and presented.

### 4.1 Evolution trajectory

Since 2006, there has been a notable and steady increase in research exploring the interplay between HIV and malaria. This trend reflects an increasing recognition within the academic community of the serious challenges posed by co-infection, especially in regions where both diseases are prevalent. Several factors have contributed to this increasing trend. One of the foremost reasons is the significant burden of HIV-malaria co-infection among pregnant women and children in sub-Saharan Africa, which has drawn substantial research interest because of its serious clinical and public health implications. In addition, the United Nations Millennium Development Goals, which were officially launched in 2000 and transitioned to the Sustainable Development Goals in 2015, included HIV and malaria control as part of the global development agenda (Fowkes et al., 2016), encouraging substantial research efforts in this field. In addition, the establishment of the Global Fund to Fight AIDS, Tuberculosis and Malaria in 2002 (Komatsu et al., 2010), the United States President’s Emergency Plan for AIDS Relief (PEPFAR) in 2003 (Goosby et al., 2012), and the President’s Malaria Initiative (PMI) in 2005 (Winskill et al., 2017) not only enhanced the delivery of HIV and malaria therapeutics but also stimulated associated scientific investigations. Furthermore, the continued growth of research capacity across the African region has fostered an increased number of locally driven field studies offering critical primary data. The combined influence of these factors has resulted in the persistent rise in research output since 2006.

The noticeable peak in publications during 2020 could be linked to the global outbreak of COVID-19. Although the pandemic severely disrupted regular healthcare delivery and research activities, it sharply pointed out the profound effects of infectious diseases on healthcare systems and the complex challenges involved in addressing co-infections. This prompted an intensified research focus on the synergistic interactions among major previously existing infectious diseases and their elevated susceptibility under systemic healthcare stress.

### 4.2 Scientific collaboration network

The United States maintains a dominant position in terms of publication volume, accounting for 41.8% of the total output. This prominent position is largely attributable to its large and continuing investment in global health programs such as PEPFAR and PMI, as well as extensive research funding from government agencies such as the National Institutes of Health. The United States also possesses world-famous research institutions, including the University of California System, Harvard University, and Johns Hopkins University. Its influence in this field is further reinforced by its crucial role in international health research networks, which is demonstrated by its high betweenness centrality. Kenya and Uganda have produced an impressive proportion of publications, representing 7.7 and 7.6% of the overall research output, respectively. This achievement demonstrates the strong research capacity developed by these two nations in the context of high HIV-malaria co-infection. This scientific advancement is significantly supported by renowned research institutes like the Kenya Medical Research Institute and Makerere University. As regions severely affected by the dual epidemics of HIV and malaria, these countries encounter considerable co-infection burdens and public health pressures, prompting a substantial amount of epidemiological and clinical research. Additionally, sustained collaborations with European and American countries play a pivotal role in producing numerous high-quality field studies.

Although international collaboration is relatively strong, cooperation among institutions appears fragmented. The fragmented nature of institutional collaboration in HIV-malaria research may yield several adverse implications. Firstly, insufficient institutional collaboration may lead to the dispersion of research efforts. Different institutions can conduct duplicate research on similar issues, thereby wasting limited scientific resources. At the same time, valuable data, samples, and clinical resources may not be efficiently integrated and shared, hindering the elucidation of the intricate mechanism of HIV-malaria interaction. Secondly, due to its interdisciplinary nature, HIV-malaria research necessitates synergistic integration among basic scientists, clinical researchers, epidemiologists, public health experts, and pharmacologists. Limited cross-institutional engagement can impede the formation and effective operation of such interdisciplinary teams and obstruct knowledge integration across disciplinary boundaries, postponing breakthroughs on critical issues. Lastly, weak collaborative networks among institutions weaken the capacity to quickly mobilize, coordinate resources, and share data to respond to public health emergencies.

Several measures are necessary to overcome the obstacles presented by limited institutional collaboration in HIV-malaria research. Initially, it is critical to establish regional or multinational collaborative research networks dedicated to priority themes related to HIV-malaria co-infection, such as immunological mechanisms and drug–drug interactions. Secondly, funding agencies should encourage inter-institutional applications for large-scale and multidisciplinary research grants. Finally, developing resource allocation systems based on artificial intelligence should be accelerated to enhance rapid-response capabilities for public health emergencies.

### 4.3 Hot research topics

#### 4.3.1 Epidemiologic study of different populations in the African region

The bibliometric analysis indicates an increasing emphasis on epidemiological studies on diverse populations in Africa. Through keyword co-occurrence analysis, “children,” “mortality,” “prevalence,” “sub-Saharan Africa,” and “Africa” were identified as high-frequency keywords. “Global health” and “mortality” are also highlighted by keyword cluster analysis as important research topics in the field. In addition, a surge in the occurrence number of keywords such as “morbidity,” “prevalence,” “pregnant women,” “child health,” “global burden,” and “public health” is revealed by keyword burst analysis, highlighting the increased interest in the epidemiological studies of HIV-malaria co-infection.

In sub-Saharan Africa, the epidemiological profile of HIV and malaria co-infection varies significantly by region. According to a meta-analysis conducted in 2021, significant regional variations were found in the prevalence of malaria among HIV-positive patients in sub-Saharan Africa. The prevalence ranged from 12.6% in the eastern region, 15.3% in the central region, and 30.2% in the western region to as high as 33.1% in the southern region, resulting in an overall pooled prevalence of 22.7% (Obebe and Falohun, 2021). Distinct variations in the epidemiological characteristics of HIV-malaria co-infection are also observed across different population subgroups, highlighting the necessity for targeted interventions customized for particular demographic groups. A 2016 meta-analysis indicated a pooled prevalence of HIV-malaria co-infection of 19%, with subgroup analyses revealing prevalence rates of 26% among adults, 12% among pregnant women, and 9% among children (Naing et al., 2016). A meta-analysis in 2022 demonstrated that HIV-positive children had a malaria prevalence of 39.4%, considerably greater than HIV-positive pregnant women at 32.3% and HIV-positive adults at 27.3% (Mirzohreh et al., 2022). These observations not only underscore the necessity for regionally tailored surveillance systems that prioritize high-risk populations but also highlight the imperative for epidemiological studies to possess both timeliness and accuracy to effectively capture the true disease burden of HIV-malaria co-infection.

HIV-malaria co-infection can lead to particularly severe clinical consequences for pregnant women and children. According to a meta-analysis, compared with individuals suffering from *Plasmodium* mono-infection, those co-infected are 2.41 times more likely to develop severe malaria (*p* = 0.001). As for children, the risk increases more significantly, with a striking 9.69 times higher likelihood of severe malaria (*p* < 0.0001). This study points out the critical necessity for early identification and integrated management of malaria and HIV co-infections, especially in pediatric populations, to diminish morbidity and mortality in endemic areas (Mahittikorn et al., 2021). In a cross-sectional study involving severely undernourished children in Congo, the prevalence of HIV and malaria co-infection was 29.2%, and this comorbidity was significantly linked to heightened mortality and negative clinical outcomes (Jacques et al., 2020). In Mozambique, pregnant women with HIV-malaria co-infection faced a higher risk of peripartum urinary tract infections than those without co-infection (*p* = 0.003). Neonates born to co-infected mothers were at a greater risk of complications (*p* = 0.020), such as stillbirth, neonatal asphyxia, and neonatal jaundice (Jaen-Sanchez et al., 2023). These findings indicate a harmful interplay between HIV and malaria, which is more intense in people with weaker immune systems. Integrated antenatal screening and enhanced pediatric care targeting co-infection could mitigate these severe outcomes.

Healthcare access significantly influences HIV-malaria co-infection epidemiology. A meta-analysis investigated the epidemiology of malaria among HIV-positive patients in sub-Saharan Africa. The results indicated that ART was linked to a significantly lower risk of malaria infection among HIV-positive individuals. Patients undergoing ART exhibited a 63% reduced likelihood of developing malaria compared to those not receiving ART (Obebe and Falohun, 2021). The protective effect of ART is also substantiated by research conducted in Cameroon, which documented a low incidence of malaria among HIV-infected patients on ART (7.9–9.4%) (Tankoua-Tchounda et al., 2024; Ebai et al., 2025). This advantage could be explained by the fact that ART strengthens the immune function in HIV-positive individuals, which reduces their susceptibility to malaria. A randomized clinical trial conducted in Malawi assessed the incidence of malaria infection in clinically stable adults living with HIV undergoing ART after the cessation of malaria chemoprophylaxis. The discontinuation of malaria prophylaxis in HIV-infected adults led to a markedly elevated clinical malaria incidence (21.4/100 person-years) in contrast to those maintaining prophylaxis with either co-trimoxazole (2.4/100 person-years) or chloroquine (1.9/100 person-years). The preventive effect of continued prophylaxis was substantial, with approximately a 90% reduction in malaria incidence. The research indicates that individuals with HIV who are stable on ART should continue malaria prophylaxis to mitigate the burden of malaria (Mungwira et al., 2022). In a cross-sectional study in Cameroon, HIV-infected children who are not receiving ART (adjusted OR = 2.2, 95% CI 1.03–4.74) or co-trimoxazole prophylaxis (adjusted OR = 9.08, 95% CI 2.33–43.46) exhibit a significantly elevated risk of malaria infection (Ngwa et al., 2024). According to these studies, in order to mitigate these risks and enhance overall health outcomes, public health strategies should place a high priority on improving access to ART and malaria chemoprophylaxis.

Individuals co-infected with asymptomatic malaria and HIV constitute a significant reservoir of infection and transmission, presenting a substantial challenge to malaria elimination efforts in endemic regions. The epidemiological investigation of asymptomatic malaria and HIV co-infection is crucial for the development of integrated interventions addressing this dual burden. The prevalence of asymptomatic malaria in HIV-infected patients exhibits marked variability attributable to regional transmission intensity, host immune condition, and access to prophylactic interventions. A cross-sectional study in Gabon revealed that the prevalence of asymptomatic *P. falciparum* infection among people living with HIV was 7.1%, markedly lower than the 33.8% among HIV-uninfected individuals (*p* < 0.01). This disparity was partially ascribed to the administration of co-trimoxazole prophylaxis and higher healthcare engagement among those infected with HIV. Patients infected with HIV and possessing CD4 cell counts below 200 cells/μL exhibited a significantly higher prevalence (*p* = 0.03) (Bouyou Akotet et al., 2018). In a region of high malaria transmission in Western Kenya, the prevalence of asymptomatic malarial parasitemia in HIV-positive and HIV-negative adults was determined employing a highly sensitive *Plasmodium* genus-specific 18S assay, revealing a positivity rate of 71% in HIV-positive individuals and 64% in HIV-negative individuals. The majority of infections identified by genus-specific 18S assay were undetectable by standard microscopy or rapid diagnostic test, underscoring the significance of molecular diagnostics to accurately define the infection burden in populations with elevated rates of submicroscopic and asymptomatic parasitemia, particularly among HIV-infected adults (Kifude et al., 2021). The mitigating effect of ART and co-trimoxazole prophylaxis can improve the co-infection burden. In a longitudinal cohort from Kenya, asymptomatic parasitemia among HIV-1-positive individuals receiving stable ART was significantly reduced in comparison to HIV-1-negative individuals (hazard ratio 0.51, *p* < 0.001). Upon follow-up, there was a notable reduction in the prevalence of asymptomatic parasitemia among the HIV patients newly commenced on ART in conjunction with co-trimoxazole (hazard ratio 0.74, *p* < 0.001) (Oyieko et al., 2023).

Multi-strain malaria infections are common in malaria endemic regions. A 2020 meta-analysis showed that patients with mixed *Plasmodium* infections and those with *P. falciparum* malaria had a similar risk of developing severe malaria (OR 0.93, 95% CI 0.59–1.44). In terms of complications, patients with mixed infections had a higher incidence of severe anemia, pulmonary complications, and multiple organ failure when compared to those with *P. falciparum* mono-infection. This research highlighted that malaria with multi-strain infections was common, resulting in severe complications among these malaria patients. However, the epidemiology of malaria with multi-strain infection among HIV-infected patients is unclear. There is a deficiency of basic and clinical research regarding the interactions between mixed *Plasmodium* infections and HIV. Therefore, research on this issue should be strengthened in the future.

#### 4.3.2 Unraveling the pathogenic mechanisms underlying HIV-malaria co-infection

The emphasis on pathogenic mechanisms in HIV-malaria co-infection reflects both a scientific priority and trends revealed by the bibliometric analysis. In the analysis of cocited references, the influential studies by Whitworth et al. (2000) and Flateau et al. (2011) are among the top three most cocited. This underscores the foundational role of immunological mechanisms. Keywords such as “antibody” and “expression” point toward research into the mechanisms of co-infection. It is critical to understand the pathogenic mechanisms in order to facilitate the development of targeted immune-based therapies and predict the progression of co-infection.

HIV infection can impair antimalarial immunity, and CD4^+^ T cell depletion is a major factor in determining malaria susceptibility. In a Chinese rhesus macaque model co-infected with simian immunodeficiency virus (SIV) and *Plasmodium cynomolgi* malaria, it was shown that the SIV and malaria co-infected animals experienced more severe malaria symptoms compared to those with malaria alone. These included prolonged malaria episodes, elevated parasitemia, aggravated anemia, increased weight loss, and raised body temperature. Co-infected animals showed fewer numbers of total CD4^+^ T cells, CD4^+^CD28^high^CD95^high^ central memory T cells, and CD4^+^CD28^low^CD95^−^ naive T cells than those infected solely with malaria. These changes in immune cell populations may be the consequence of disrupted immune activation and accelerated turnover of CD4^+^ T cells in the co-infected animals (Liu et al., 2019). SIV-malaria co-infection in animal models has been shown to result in increased severity of malaria symptoms. However, the precise clinical manifestations and underlying immunopathological mechanisms may differ between species. Further clinical studies in humans are needed to clarify whether SIV-malaria and HIV-malaria co-infections result in differential symptoms or pathogenesis.

Clinical studies have demonstrated that low CD4^+^ T cell counts are strongly associated with increased malaria susceptibility and more severe clinical outcomes in HIV-infected individuals. A meta-analysis indicated that a CD4^+^ count of below 200 cells/μL was associated with an increased risk of malaria infection in pregnant women with HIV (OR = 1.5, 95% CI = 1.1–1.9) (Mirzohreh et al., 2022). HIV-infected children also demonstrated a 3.09-fold elevated risk of malaria infection when CD4^+^ counts were below 200 cells/μL compared to children who had higher CD4^+^ counts (Ngwa et al., 2024). This increase in malaria risk is related to the immunosuppression caused by HIV, which reduces the host’s ability to control malaria parasite multiplication, therefore increasing the frequency and severity of malaria infections (Obeagu et al., 2024). Compared to children solely infected with HIV, this immunosuppressive condition was evident in hematological profiles, with substantial decreases in red blood cells (*p* < 0.001), hemoglobin (*p* = 0.0016), white blood cells (*p* = 0.002), neutrophil count (*p* < 0.001), and platelet count (*p* = 0.0164) in those co-infected with HIV and malaria (Ngwa et al., 2024). Additionally, a cross-sectional study conducted in Southwest Ethiopia reported that, among HIV-malaria co-infected patients on ART, there was a significantly higher prevalence of anemia (63.4%) when compared to patients with HIV mono-infection on ART (36.6%). The co-infected patients had significantly lower red blood cell counts, hemoglobin concentrations, and hematocrit levels than the patients with HIV mono-infection (*p* < 0.001). CD4 counts showcased a positive correlation with red blood cell counts, hemoglobin concentrations, and hematocrit levels in HIV-malaria co-infected patients. This could be due to impaired erythropoietin production driven by HIV-related myelosuppression and hemolysis caused by *P. falciparum* (Ejigu et al., 2022). In summary, HIV-induced immunosuppression dramatically accelerates malaria pathogenesis and hematological deterioration, underscoring the necessity of integrated immune and hematologic monitoring in co-infected patients.

Some evidence has indicated that HIV infection may also impair antimalarial humoral responses. An animal experiment revealed that SIV-malaria co-infected subjects exhibited a weakened B cell response to *P. cynomolgi* malaria and generated reduced levels of *P. cynomolgi*-specific antibodies (Liu et al., 2019). A cohort study in Mozambique revealed that HIV-positive mothers exhibited broadly reduced levels of IgG antibodies and their subclasses against various *P. falciparum* antigens in peripheral blood. Moreover, maternal antibody levels exhibited a strong positive correlation with cord blood antibody levels against all the antigens and subclasses. Maternal HIV infection diminished the antibody levels in cord blood against some antigens of *P. falciparum*, manifested by a decrease of IgG to exported protein 1 and merozoite surface proteins (MSP) 5 by 3.84 and 1.47%, respectively; IgG1 to MSP2 and reticulocyte-binding-homolog-4.2 by 9.09 and 3.12%, respectively; and IgG4 to MSP1_42_ by 1.91%. The reduction in antibody levels was partially attributable to the compromised transplacental transfer of antibodies resulting from HIV infection and the changes in maternal antibody concentrations, which predominantly affect cord blood levels. This study elucidated the heightened susceptibility to malaria in infants exposed to HIV and those born from mothers with placental malaria (Alonso et al., 2021). Research has discovered that in individuals with HIV-malaria co-infection, there is a significant expansion of atypical memory B cells, which is closely associated with compromised humoral immunity against malaria. Although these atypical memory B cells are antigen-experienced and undergo somatic hypermutation, they lack CD27, the classical memory B cell marker, and express higher levels of inhibitory receptors, including Fc-receptor-like-4. Functionally, atypical memory B cells have diminished capacities to respond to antigenic stimulation *in vitro*, suggesting immune dysfunction (Moir et al., 2008). A study on HIV-infected adults in Kenya found that HIV infection was linked to a significant change in the composition of B cell subsets, even though there was no apparent reduction in serum antibody levels against *P. falciparum*’s apical membrane antigen 1. The study demonstrated an increase in total atypical and total activated memory B cells and a decrease in resting memory B cells and naive B cells. Instead of atypical and activated cells actively expanding, this increase was mainly caused by the depletion of naive and resting memory B cells (Frosch et al., 2017). A prospective study in Rwandan adults revealed that individuals co-infected with HIV and malaria had significantly higher frequencies of atypical memory B cells in comparison to those with malaria alone. This was accompanied by a marked reduction in both the breadth and magnitude of antibody responses to various *P. falciparum* antigens. These findings support that the increase of atypical memory B cells is indicative of a dysregulation of the B cell compartment and a poorer capacity for effective antigen-specific humoral responses. Impaired B cell function may contribute to increased susceptibility to malaria among people with HIV (Subramaniam et al., 2015). These findings indicate that HIV infection causes severe disruption of the humoral immune response toward malaria, resulting in diminished antibody production and altered distribution of B cell subsets. Future studies should explore the mechanisms through which HIV disrupts B cell function and antibody-mediated immunity against malaria.

Interestingly, the sequence of infection critically influences immunological outcomes and disease progression in HIV and malaria co-infection. This was thoroughly elucidated utilizing a Chinese rhesus macaque model of HIV-malaria co-infection. When malaria follows established SIV infection, a remarkable increase in CD4^+^CD28^high^CD95^high^ central memory T cells occurs, accompanied by enhanced SIV-specific T cell responses. The subsequent malaria maintained the repertoire diversity of SIV-specific T cell receptors and produced new SIV-specific T cell clonotypes for monitoring antigenic variation, leading to higher survival rates in SIV-infected animals. On the other hand, pre-existing malaria promoted SIV disease progress by producing more CD4^+^CCR5^+^ T cells for SIV replication (Liu et al., 2022). This study emphasizes the importance of infection sequence for disease outcomes. Malaria after HIV infection seems to provide immunological benefits by increasing central memory T cells and enhancing viral immune control. Malaria before HIV infection may accelerate disease progression by increasing the pool of susceptible target cells and reducing the host’s immune recovery capacity.

In addition, studies on many biomarkers show that HIV-malaria co-infection results in significant immune activation and inflammation dysregulation, deepening our understanding of its pathogenic mechanisms and providing valuable biomarkers for clinical diagnosis, prognostic assessment, and individualized therapeutic strategies. A prospective cross-sectional study in Mozambique demonstrated that HIV-infected patients with malaria exhibited significantly elevated levels of myeloperoxidase (MPO), an indicator of neutrophil activation, and sCD25, an indicator of T-cell activation, in comparison to both malaria patients without HIV infection and HIV-infected patients without malaria, indicating heightened activation of neutrophils and T-cells in those with co-infection. Moreover, co-infected patients showcased boosted levels of granzyme B and CX3CL1, suggesting increased activation of CD8^+^ T-cells and effector memory T-cells, as well as TIM-3, which denotes some degree of T-cell exhaustion. The study underscores the association between immune activation and immune exhaustion in severe *falciparum* malaria, especially in relation to T-cell responses in patients with HIV-malaria co-infection (Otterdal et al., 2018). Another study from the same team discovered that IL-18 and IL-18 binding protein (IL-18 bp) were significantly upregulated during malaria, with the highest levels in malaria patients co-infected with HIV and experiencing severe malaria. This study also proved that IL-27 (upregulated in patients with HIV and malaria co-infection) markedly stimulated the release of IL-18 bp (an endogenous antagonist of IL-18) from endothelial cells *in vitro*, and particularly, this presumably anti-inflammatory action was mitigated by hemozoin. These findings provided novel insights into the immune regulatory network associated with HIV and malaria co-infection, deepening our understanding of immune regulation in the context of co-infection (Otterdal et al., 2021). A case–control study by Chukwuagwu et al. determined that pregnant women co-infected with HIV and malaria presented dramatically elevated levels of IL-6, IFN-γ, and TNF-α in comparison to HIV-negative pregnant and nonpregnant control subjects. The cytokine dysregulation indicated an active inflammatory response and diminished cellular immunity. Regular assessment of these cytokines may function as a supplementary diagnostic marker in HIV-positive pregnant women with malaria co-infection, particularly in regions with high endemic transmission (Chukwuagwu et al., 2020). In placental blood collected from Kenyan women, MPO and proteinase 3 were substantially increased in instances of placental malaria and, more dramatically, in cases of placental malaria/HIV co-infection, correlating with placental parasite density and the accumulation of hemozoin-laden leukocytes (Sarr et al., 2021). Further studies on dynamic regulation and functional effects of these biomarkers may improve the precise management of co-infected patients and the translation of immunological findings to clinical practice.

In a pilot study, the rhesus macaque model was employed to simulate *Plasmodium fragile* co-infection in SIV infection that was treated with ART. It was discovered that *P. fragile* co-infection was linked to elevated levels of inflammatory cytokines, such as monocyte chemoattractant protein 1. *P. fragile* co-infection was also correlated with boosted neutrophil elastase levels, an indicator of neutrophil extracellular trap formation, alongside substantial reductions in Cathepsin G, a marker of neutrophil degranulation, suggesting a potential alteration in the neutrophil function in co-infection. Moreover, *P. fragile* co-infection elevated the levels of plasma indicators of gastrointestinal barrier permeability (intestinal fatty acid-binding protein) and microbial translocation (lipopolysaccharide binding protein and sCD14). Multivariate analysis of variance indicated significant correlations among indicators of gastrointestinal disorder, clinical indicators of SIV and *P. fragile* infection, and neutrophil frequency and function. These results indicated that neutrophil-mediated inflammation and gastrointestinal dysfunction may exacerbate the SIV/*P. fragile* co-infection pathogenesis (Nemphos et al., 2024). This study lays important scientific groundwork for managing HIV-malaria co-infection and developing targeted therapeutic strategies in co-endemic areas.

A comprehensive investigation of the immunopathogenic interaction between HIV and malaria should be the main focus of future research, emphasizing clarifying the temporal sequence of co-infection and its consequences for host immune modulation. It is critical to develop targeted immunotherapeutic strategies that tackle specific pathogenic mechanisms in co-infected populations, especially by augmenting central memory T cell responses and maternal antibody transfer. Moreover, early risk stratification benefits from the discovery and application of novel biomarkers. Finally, clarifying mechanistic details and confirming novel therapeutic approaches require the development of animal models that accurately replicate human co-infection.

#### 4.3.3 Exploring prevention and treatment strategies of HIV-malaria co-infection

Co-infection prevention and treatment measures for HIV and malaria have garnered a lot of research interest. Keyword cluster analysis reveals that “antiretroviral therapy” and “intermittent preventive treatment” are important areas of interest, highlighting the growing importance of combined preventive measures. Moreover, cocited studies with high influence emphasized the effectiveness of co-trimoxazole prophylaxis, antiretroviral medications, and ITNs in co-infected individuals. Overall, exploring effective management of HIV-malaria co-infection is crucial for alleviating disease burden and improving patient outcomes.

Current management practices emphasize the necessity of integrated malaria prevention and control strategies for people living with HIV, including long-lasting ITNs, indoor residual spraying, timely and accurate diagnosis, and effective treatment. In regions where malaria transmission is high, the 2024 WHO guidelines for malaria recommend several preventive approaches: providing pregnant women with intermittent preventive treatment with sulfadoxine-pyrimethamine (SP), administering perennial malaria chemoprevention with SP to children, and offering seasonal malaria chemoprevention to children using a combination of SP and amodiaquine. For the treatment of uncomplicated *P. falciparum* malaria, the guidelines advise prompt diagnosis and a three-day regimen of artemisinin-based combination therapy, such as AL, DHA-PPQ, and artesunate-amodiaquine (WHO, 2024b). Dolutegravir combined with a nucleoside reverse transcriptase inhibitor backbone is the current preferred first-line ART regimen. In the neonatal population, a raltegravir-based regimen is recommended as the preferred first-line therapeutic option (WHO, 2021). Co-infection management demands particular attention to potential drug–drug interactions between antimalarial therapies, ART, and prophylactic medications used for opportunistic infections. For example, chemoprevention with SP is contraindicated in HIV-positive pregnant women or children who are receiving co-trimoxazole prophylaxis (WHO, 2024b). Numerous studies have shown that effective prevention and timely management of HIV-malaria co-infection can significantly reduce the incidence of severe malaria (Obebe and Falohun, 2021; Mungwira et al., 2022). Overall, these strategies have greatly improved clinical outcomes and enhanced the quality of life for people with HIV-malaria co-infection.

As part of standard care for HIV-positive individuals, daily co-trimoxazole prophylaxis is recommended to prevent opportunistic infections. It confers some degree of protection against malaria due to its antifolate activity. A pragmatic randomized controlled trial indicated that co-trimoxazole exhibited favorable safety and high patient compliance, rendering it a significant and practical option for malaria prevention in HIV-positive pregnant women (Manirakiza et al., 2021). However, the antimalarial efficacy of daily co-trimoxazole is compromised by cross-resistance with SP, attributable to their common antifolate mechanism. Due to the increasing resistance of *P. falciparum* to SP, alternative preventive strategies are required. DHA-PPQ, a long-acting artemisinin-based combination therapy with a distinct mechanism of action, has demonstrated potential as an intermittent preventive treatment during pregnancy in HIV-positive women.

Recent evidence from two randomized controlled trials demonstrated that adding DHA-PPQ to daily co-trimoxazole significantly reduced malaria infection risk in HIV-positive pregnant women. In a randomized, placebo-controlled, double-blind trial carried out in regions with high-grade SP resistance in Kenya and Malawi, the incorporation of monthly intermittent preventive treatment with DHA-PPQ to daily co-trimoxazole prophylaxis significantly diminished the cumulative risk of any malaria infection during pregnancy or delivery among pregnant women living with HIV receiving dolutegravir-based ART (RR 0.45, 95% CI 0.30–0.67, *p* = 0.0001). The intervention also decreased the incidence of any malaria infection (incidence RR 0.32, 95% CI 0.22–0.47, *p* < 0.0001). The intervention exhibited a favorable safety profile, with no substantial rise in serious adverse events in either mothers or infants and no difference in the risk of adverse pregnancy outcomes or mother-to-child HIV transmission compared to the co-trimoxazole plus placebo group (Barsosio et al., 2024). In another randomized, double-blind, placebo-controlled trial conducted in Gabon and Mozambique, researchers evaluated the safety and efficacy of intermittent preventive treatment with DHA-PPQ in pregnant women who suffered from HIV and were taking daily co-trimoxazole prophylaxis and ART. The results indicated that the daily co-trimoxazole plus DHA-PPQ group had a reduced incidence of clinical malaria during pregnancy compared to the placebo plus DHA-PPQ group (RR 0.12, 95% CI 0.03–0.52, *p* = 0.045). Furthermore, a post-hoc analysis showed that the intervention group had a significant reduction in the composite outcome of overall malaria infection in the intervention group (RR 0.48, 95% CI 0.27–0.84, *p* = 0.010). The incidence of severe adverse events and negative pregnancy outcomes (miscarriages, stillbirths, premature births, and congenital anomalies) was similar across both groups. The results provide further evidence that DHA-PPQ is a safe and effective method for preventing malaria in HIV-infected pregnant women concurrently receiving co-trimoxazole and ART (Gonzalez et al., 2024). The Cochrane systematic review indicated that DHA-PPQ administered with daily co-trimoxazole was efficacious in preventing malaria infection in HIV-positive pregnant women compared with co-trimoxazole alone. High-certainty evidence indicated that this combination markedly decreased the incidence of placental malaria, as determined through histopathologic evaluation (RR 0.67, 95% CI 0.50–0.90). Evidence of low certainty revealed that this combination did not elevate the risk of mother-to-child HIV transmission (RR 1.54, 95% CI 0.26–9.19) or drug-related gastrointestinal adverse events (RR 1.42, 95% CI 0.51–3.98) (Pons-Duran et al., 2024). These findings suggest that DHA-PPQ, when co-administered with co-trimoxazole, could be a viable option for malaria prevention in HIV-infected pregnant women, especially in the context of transitioning to dolutegravir-based ART regimens.

However, the most susceptible demographic, specifically children who are concurrently exposed to HIV and at risk of malaria, are presently absent of effective chemopreventive measures. These children are excluded from malaria chemoprevention with SP due to their simultaneous daily administration of co-trimoxazole prophylaxis. González et al. propose that drawing on the lessons learned from malaria prevention strategies in HIV-positive pregnant women, similar methods should be implemented for HIV-exposed children. It is essential to conduct clinical trials on alternative antimalarial medications that demonstrate robust efficacy and safety and ensure compatibility with the standard co-trimoxazole prophylaxis regimen. To guarantee that global health interventions effectively address the needs of all vulnerable populations, particularly HIV-exposed children, further research and policy reforms are imperative (González et al., 2024).

In addition, some studies showed the impact of ART regimens and co-trimoxazole prophylaxis on malaria outcomes. In Nigeria, a pilot cross-sectional study found that individuals with HIV receiving first-line combination ART had a lower malaria parasite burden than antiretroviral-naive individuals with HIV. Patients receiving tenofovir disoproxil fumarate/lamivudine/efavirenz exhibited a lower proportion of malaria antigenemia (6.67%) than those on zidovudine/lamivudine/nevirapine (16.67%). According to the study, in regions where malaria is endemic, tenofovir disoproxil fumarate/lamivudine/efavirenz may be used as an alternate first-line antiretroviral regimen (Onukak et al., 2024). A longitudinal cohort study conducted in Western Kenya evaluated the effects of initiating ART (lamivudine, tenofovir disoproxil fumarate, and dolutegravir) alongside co-trimoxazole on asymptomatic malaria parasitemia in HIV-1-positive individuals. The results indicated that ART/co-trimoxazole initiation substantially decreased parasitemia, with an 85.8% reduction within 1 week and a further decline of 96% following 2 weeks. There was an increase in CD4^+^ T cell levels after the initiation of ART and co-trimoxazole treatment among newly diagnosed HIV-positive individuals. However, no significant correlation was observed between CD4^+^ T cell levels and parasitemia. This implies that the reduction in parasitemia reported in HIV-positive individuals receiving treatment can be attributed to the effects of co-trimoxazole rather than immune reconstitution linked to ART. These findings underscore the substantial effect of co-trimoxazole in diminishing asymptomatic malaria in HIV-1-infected individuals, showcasing the necessity for continuous monitoring of co-trimoxazole efficacy and the potential for resistance development (Kifude et al., 2022). Collectively, these results underscore the importance of selecting optimal ART regimens and maintaining effective chemoprophylaxis to mitigate malaria risk among HIV-infected individuals in endemic areas, highlighting the necessity for continuous monitoring and adaptation of prevention strategies in clinical practice.

Transmission control measures, such as ITNs, play a key role in HIV-malaria co-infection prevention. For people living with HIV in malarious settings, ITNs are simple and effective prophylactics that have been widely adopted and recommended. By a mathematical model, Mohammed-Awel et al. investigated HIV and malaria co-infection spreading and the effects of ITNs on malaria treatment. The results showed that ITNs combined with malaria treatment reduced malaria and HIV infections and the costs of disease control. Under specific circumstances, the implementation of optimal malaria treatment and ITNs could eliminate malaria from the population and substantially decrease HIV prevalence, from 26% without intervention to 8.47% with intervention, as indicated by numerical simulations that employed optimal control strategies (Mohammed-Awel and Numfor, 2017). An unmatched case–control study in Northeast Ethiopia demonstrated that non-use of ITNs was significantly associated with HIV-malaria co-infection (adjusted OR = 6.21, 95% CI 2.74–14.11) (Yibeltal et al., 2020). However, despite the potential of ITNs in diminishing HIV-malaria co-infection, their practical efficacy is significantly affected by factors such as the maintenance condition of the nets and proper usage practices. A cross-sectional study in Nigeria indicated that although 83% of HIV-positive individuals owned an ITN, only 48% of those with ITNs claimed to possess optimal nets, and merely 45.5% of patients stated they slept under the net daily in the past week. These findings highlight the essential requirement for annual evaluations of ITN conditions and timely replacement of old and worn-out ITNs to maintain their protective efficacy (Afolabi et al., 2023).

Overall, effective prevention of HIV-malaria co-infection necessitates a multifaceted strategy involving the combination of ART, co-trimoxazole prophylaxis, intermittent preventive treatment, and ITNs. These strategies, tailored to the distinct requirements of HIV-infected individuals, can substantially reduce the health risks associated with malaria co-infection. Furthermore, as evidence continues to accumulate, it is essential to refine these interventions and adjust them to the evolving epidemiological landscape of HIV-malaria co-infection.

### 4.4 Research frontiers

#### 4.4.1 Elucidating interactions between antimalarial and antiretroviral drugs

In recent years, the detailed understanding of interactions between antimalarial and antiretroviral drugs has emerged as an important focus of research, stemming from the immediate demands of clinical practice. With ART being widely used globally (especially in sub-Saharan Africa) and artemisinin combination therapy being the first-line treatment for malaria, millions of co-infected patients are faced with long-term multidrug regimens. Furthermore, with the development of HIV-malaria treatment protocols, more and more attention has been directed toward optimizing therapeutic strategies to improve the safety and efficacy of co-infection treatments. This is consistent with the bibliometric analysis results. In particular, the work by Kwenti (2018) has a prominent burst, lasting until 2024. This article underscored the critical importance of understanding drug–drug interactions in patients receiving antimalarials and antiretroviral medications concurrently. Keyword cluster analysis also confirmed these findings by identifying “drug resistance” as a key theme.

In areas with a high prevalence of HIV and malaria co-infection, the pharmacokinetic interactions between antimalarial and antiretroviral drugs present substantial clinical challenges. The study by Nyangulu et al. highlighted the therapeutic efficacy of AL among HIV-positive adults undergoing ART in Malawi. Participants on efavirenz-based ART exhibited lower lumefantrine levels compared to those on other regimens, with diminished concentrations correlating with an increased incidence of malaria infections post-treatment. These findings underscore the significance of comprehending drug–drug interactions in the context of frequently co-occurring diseases (Nyangulu et al., 2023). This study also indicates that lower lumefantrine levels may facilitate the emergence and spread of antimalarial drug resistance, as sub-therapeutic drug exposure enables the survival and transmission of drug-resistant parasites. In this study, most HIV-infected individuals concurrently received co-trimoxazole prophylaxis. This significantly reduced the incidence of clinical malaria, thereby diminishing the size of the parasite reservoir exposed to subtherapeutic lumefantrine levels and consequently mitigating the risk of resistance dissemination. Therefore, the authors caution that discontinuation of co-trimoxazole prophylaxis may amplify the impact of these drug–drug interactions by sustaining selection pressure for resistant strains, thereby posing a substantial public health threat regarding the emergence and spread of antimalarial resistance (Nyangulu et al., 2023). In Ugandan children with HIV on efavirenz-based ART, the therapeutic efficacy of AL was examined, comparing the standard 3-day regimen to an extended 5-day regimen. The findings indicated that the prolonged 5-day regimen markedly enhanced the cumulative exposure to artemether, dihydroartemisinin, lumefantrine, and desbutyl-lumefantrine relative to the 3-day regimen (all *p* < 0.001) in the context of concurrent efavirenz administration, and this exposure was comparable to that in children without HIV receiving the standard 3-day regimen. The research showed that the prolonged 5-day AL treatment course counterbalanced the inadequate exposure to AL, thereby providing a safe and potentially more effective treatment option for the treatment of malaria in children with HIV using efavirenz-based ART (Whalen et al., 2025). Therefore, extending the duration of AL administration can compensate for reduced drug exposure and may mitigate the emergence and spread of antimalarial drug resistance.

DHA-PPQ is highly efficacious for malaria chemoprevention during pregnancy. Hong and colleagues investigated the effect of pregnancy and efavirenz-based ART on the unbound fraction of piperaquine, which is crucial for its antimalarial efficacy. In pregnant women receiving efavirenz-based ART, the unbound piperaquine fraction was 23% (*p* < 0.01) higher than in postpartum women not receiving efavirenz, potentially due to efavirenz displacing piperaquine from plasma proteins. The findings imply that the effectiveness of piperaquine in preventing malaria may not be significantly affected by the reduction in total piperaquine exposure due to the elevated level of unbound piperaquine. Additional research during the terminal elimination phase (e.g., on day 28 post-dose) would enhance the characterization of the unbound piperaquine exposure throughout the complete dosing interval, thereby elucidating the overall efficacy of piperaquine for malaria chemoprevention in this specific population (Hong et al., 2023). The emergence of dolutegravir as the preferred first-line antiretroviral agent has transformed the landscape of these drug interactions. In contrast to efavirenz, dolutegravir exhibits more favorable interaction profiles with antimalarials. Compared with efavirenz-based ART, co-administration of DHA-PPQ and dolutegravir-based ART resulted in a 57% increase in overall piperaquine exposure. The results indicate that the effectiveness of DHA-PPQ will be preserved in pregnant women receiving dolutegravir (Banda et al., 2022a).

Protease inhibitors also demonstrate a beneficial interaction pattern with antimalarials, typically improving exposure to antimalarial medications. Usman et al. assessed the impact of atazanavir-ritonavir (ATVr) on the pharmacokinetics of lumefantrine in patients living with HIV in Nigeria. ATVr markedly elevated the mean concentration of lumefantrine on day 7 by 179.88% (*p* = 0.016) and overall lumefantrine exposure by 49.68%, although the latter was not statistically significant (*p* = 0.224). This proves that AL is efficacious in treating malaria in patients undergoing an ATVr-based regimen (Usman et al., 2021). Similarly, another study confirmed that lopinavir-ritonavir enhanced lumefantrine exposure by inhibiting CYP3A4. In addition, the study also found that recurrent infections exhibited notable selection for the wild-type alleles *pfmdr1 N86* and *pfcrt K76*, which are associated with diminished susceptibility to lumefantrine. Drug-resistant parasites demonstrated tolerance to lumefantrine concentrations approximately 3.5 times greater than those of sensitive strains. This is the inaugural population pharmacokinetic model of lumefantrine in HIV-infected children, illustrating the selection for reduced lumefantrine susceptibility (Kay et al., 2023).

Furthermore, some studies explored the impact of antimalarial drugs on antiretrovirals. The co-administration of AL or artesunate/amodiaquine resulted in a 10.6% (95% CI 4.09–34.5%) and 26.4% (95% CI 14.3–51.4%) increase in dolutegravir clearance, respectively. Simulations indicated that the trough concentrations of dolutegravir, whether administered alone or in conjunction with AL or artesunate/amodiaquine, remained above the protein-adjusted IC_90_ (0.064 mg/L) of dolutegravir in over 99% of the subjects. The above results demonstrate that dose modifications of dolutegravir are unnecessary for patients receiving standard 3-day treatment regimens of AL or artesunate/amodiaquine (Kawuma et al., 2021). Moreover, Banda and coworkers examined the effect of DHA-PPQ for malaria intermittent preventive treatment on dolutegravir exposure in pregnant women living with HIV. The concomitant administration of DHA-PPQ and dolutegravir yielded a 30% increase in the overall exposure of dolutegravir (AUC_0–24_) (90% CI 1.11–1.52) and a 31% elevation in Cmax (90% CI 1.13–1.51), respectively. The trough (C_24_) concentration of dolutegravir rose by 42% (90% CI 1.09–1.85). The integrated therapies were well tolerated, with no notable side effects recorded. The elevated dolutegravir exposure may result from the inhibition of efflux transporters by DHA-PPQ (Banda et al., 2022b).

In particular, the toxicities linked to the simultaneous administration of AL and ATVr-based regimens in HIV-positive patients were evaluated. Usman et al. discovered that co-administration of AL with ATVr led to a significant elevation in the QTc interval from 0.4079 ± 0.008 to 0.4215 ± 0.007 s (*p* = 0.008), while no apparent alteration was observed in the control group (*p* = 0.962). Although these values were maintained within a normal range, this finding necessitates caution when co-administering these medications, especially in patients with cardiac risk factors. However, the plasma levels of creatinine, hemoglobin, aspartate aminotransferase, and alanine aminotransferase did not exhibit any significant alteration (*p* > 0.05) in either study arm at the post-dose compared to the pre-dose of AL. Collectively, the combined administration of AL with an ATV-based regimen may be cardiotoxic, but it is not linked to clinically significant renal, hematological, or hepatic toxicities (Usman et al., 2022).

The intricate interactions between antimalarial and antiretroviral drugs have been systematically elucidated by recent research, offering essential evidence for clinical decision-making in patients co-infected with HIV and malaria. According to current research, ART regimens based on dolutegravir or protease inhibitors exhibit superior interaction profiles with artemisinin-based combination therapies (AL or DHA-PPQ). In contrast, efavirenz-based regimens necessitate modifications in treatment duration or dosage to address suboptimal drug exposure. Nevertheless, substantial obstacles persist with respect to drug resistance and safety. The emergence of lumefantrine-tolerant parasites is particularly concerning for pediatric populations. Furthermore, the clinically mild QTc prolongation that results from the co-administration of ATVr and AL should be addressed with caution in patients who have cardiac risk factors. These results underscore the necessity of comprehensive monitoring of cardiac safety, resistance markers, and therapeutic efficacy in co-infected populations. Future studies should concentrate on determining the clinical significance of interactions with newly developed antiretroviral and antimalarial drugs, optimizing dosing schedules for susceptible populations, and evaluating the potential long-term effects of these interactions on therapeutic efficacy and antimalarial resistance in endemic areas.

#### 4.4.2 Deciphering the malaria vaccine responses in HIV-infected individuals

The shift of malaria vaccines from development to implementation drives the emergence of research on malaria vaccination responses in individuals with HIV as a major frontier. The optimization of immunogenicity and safety profiles in the context of HIV infection has become a focus of scholarly attention as global malaria vaccine research continues to progress. The cocited reference, Kwenti (2018), exhibits a substantial increase in citations extending into 2024. This paper emphasized the urgent requirement to create innovative, safer, and more economical malaria vaccines tailored to the unique immunological challenges presented by HIV-malaria co-infection. Through keyword cluster analysis, “vaccine” is also identified to be a major theme.

Recent research on the RTS, S/AS01 malaria vaccine and PfSPZ Vaccine has provided novel insights into the immunological mechanisms of malaria vaccines in HIV-positive populations. The pre-erythrocytic RTS, S/AS01 vaccine comprises portions of the *P. falciparum* circumsporozoite protein (PfCSP) and has demonstrated safety in HIV-infected children. A randomized, double-blind, controlled trial conducted in Kenya revealed that HIV-positive children with WHO stage 1 or 2 disease exhibited comparable safety profiles between RTS, S/AS01 and rabies vaccine recipients, with serious adverse events (SAEs) occurring in 41.4% (95% CI 31.6–51.8) and 36.6% (95% CI 27.3–46.8) of participants, respectively (RR 1.1, 95% CI 0.8–1.6) (Otieno et al., 2016). The study indicated that RTS, S/AS01 was well tolerated in children with WHO clinical stage 1 or 2 HIV disease, in conjunction with high antiretroviral and co-trimoxazole administration (Otieno et al., 2016). A subsequent large phase III randomized controlled trial conducted across seven sub-Saharan African nations corroborated these findings. The trial revealed that SAEs were observed in 92.2% of HIV-positive RTS, S/AS01 recipients and 87.5% of the comparator (meningococcal or rabies) vaccine recipients, indicating that the safety profile of the RTS, S/AS01 vaccine in children infected with HIV was comparable to that of the comparator vaccines (Otieno et al., 2020). While these findings support the safety of the vaccine in HIV-infected individuals, immunogenicity data demonstrated that the geometric mean antibody concentrations of anti-PfCSP in RTS, S/AS01-vaccinated HIV-infected children (193.3 EU/mL) were significantly lower than those in RTS, S/AS01-vaccinated controls with unknown or negative HIV status (491.5 EU/mL) (*p* = 0.0001) (Otieno et al., 2020). Although RTS, S/AS01 was shown to be well-tolerated and immunogenic in HIV-positive children, HIV infection may hinder humoral responses, potentially diminishing vaccine efficacy.

The *P. falciparum* sporozoites (PfSPZ) vaccine trials further illuminate the immunological disparities between HIV-positive and HIV-negative individuals. Based on whole, radiation-attenuated PfSPZ, the PfSPZ vaccine is a promising candidate for a malaria vaccine that targets the pre-erythrocytic stage. A randomized, double-blind, placebo-controlled trial evaluated the safety and protective efficacy of the PfSPZ Vaccine in HIV-positive and negative Tanzanian adults. The vaccine was determined to be safe and well-tolerated among HIV-positive participants, with no solicited adverse events reported. However, the vaccine efficacy against controlled human malaria infection was 80% in HIV-negative vaccinees (*p* = 0.012), but no protection was observed in HIV-positive vaccinees. Serum from HIV-negative vaccinees exhibited markedly higher inhibition of PfSPZ invasion of hepatocytes *in vitro* and enhanced antibody-dependent complement deposition and Fcγ3B binding by anti-PfCSP antibodies in comparison to HIV-positive vaccinees. These findings emphasize the critical role of functional antibodies in mediating vaccine-induced protection and underscore the necessity for more effective vaccine regimens or adjuvants to address the immunological deficiencies linked to HIV infection (Jongo et al., 2024). In addition, protein microarray analysis revealed that immunization with the PfSPZ vaccine elicited focused IgG and IgM responses to the PfCSP and *P. falciparum* merozoite surface protein 5 (PfMSP5), with no notable difference in response patterns between HIV-positive and negative individuals. PfMSP5 was recognized as a highly immunogenic target, exhibiting robust reactivity with IgM antibodies. The study also demonstrated that PfMSP5 is expressed in the apical complex and on the surface of sporozoites, implying its potential as a novel antigen for future malaria vaccines. This finding is particularly significant as it highlights the necessity of investigating underutilized antigens in vaccine development (Tumbo et al., 2024).

Briefly, the results of these studies provide critical insights into the intricate relationship between HIV infection and malaria vaccine responses. The safety of both RTS, S/AS01 and PfSPZ vaccines in HIV-positive individuals supports their prospective application in mass vaccination programs without necessitating HIV screening. However, the diminished immunogenicity and protective efficacy in HIV-positive populations highlight the need for optimized vaccine strategies. Subsequent research should focus on augmenting vaccine-induced immune responses through improved adjuvants, alternative dosing regimens, or the inclusion of novel antigens such as PfMSP5. Additionally, tackling the immunological obstacles presented by HIV co-infection, such as boosting functional antibody responses, will be essential for developing effective malaria vaccines that benefit both HIV-positive and negative populations in endemic areas.

## 5 Limitations

The current study presents a data-driven investigation of cooperative efforts, research themes, and prospective developmental trends in HIV and malaria interactions. Nonetheless, constraints are inevitable. Initially, the information was compiled solely from WOSCC, disregarding non-English publications, possibly resulting in publication bias. Therefore, it is plausible that the outcomes of the present study are not all-encompassing. However, any potentially overlooked factors are trivial and have a negligible influence on the results. Moreover, the personal perspectives of different scholars in choosing keywords can contribute to prejudice in hotspots and trend evaluation. The common practice of citing reviews rather than original studies, coupled with the influence of release time, may also cause a bias in citation frequency. In addition, the most recent publications from 2025 were not included in this analysis, which narrowly assessed data up to 2024. Furthermore, this study delineated the discussion on research hotspots, frontiers, and trends into five primary subsections, potentially restricting our discussion and inadequately encompassing the comprehensive landscape of HIV and malaria interaction research. Despite these limitations, as the inaugural research into HIV and malaria interactions utilizing bibliometric and visualization techniques, our study provides a comprehensive analysis of important subjects and trends in this field, offering valuable insights into current and prospective research efforts.

## 6 Conclusion

This research utilizes CiteSpace to critically evaluate academic papers concerning interactions between HIV and malaria, a topic garnering increasing global interest. The annual publication output has generally fluctuated upward from 1995 to 2024. The United States has exhibited exceptional productivity in this research domain, positioning itself as the foremost nation. Improving cooperation among transnational research institutions is essential. Based on examinations of keywords and cocited references, the predominant hot topics in this area concentrate on the epidemiological study of various populations in the African region, pathogenic mechanisms underlying HIV-malaria co-infection, and strategies for preventing and treating HIV and malaria co-infection. The research frontiers predominantly center around elucidating the interactions between antimalarial and antiretroviral drugs and deciphering the malaria vaccine responses in HIV-infected individuals. These findings guide future research concerning HIV and malaria interactions and equip scholars with a thorough comprehension of developments in this area.

## Data Availability

The original contributions presented in the study are included in the article/Supplementary material, further inquiries can be directed to the corresponding authors.
