# Supplementary figures and images for: Unraveling the HIV-malaria interactions: a bibliometric analysis of global research trends and emerging insights
Source: Front Microbiol. 2025 Aug 29;16:1622769. doi: 10.3389/fmicb.2025.1622769 (PMC12426043; doi:10.3389/fmicb.2025.1622769)

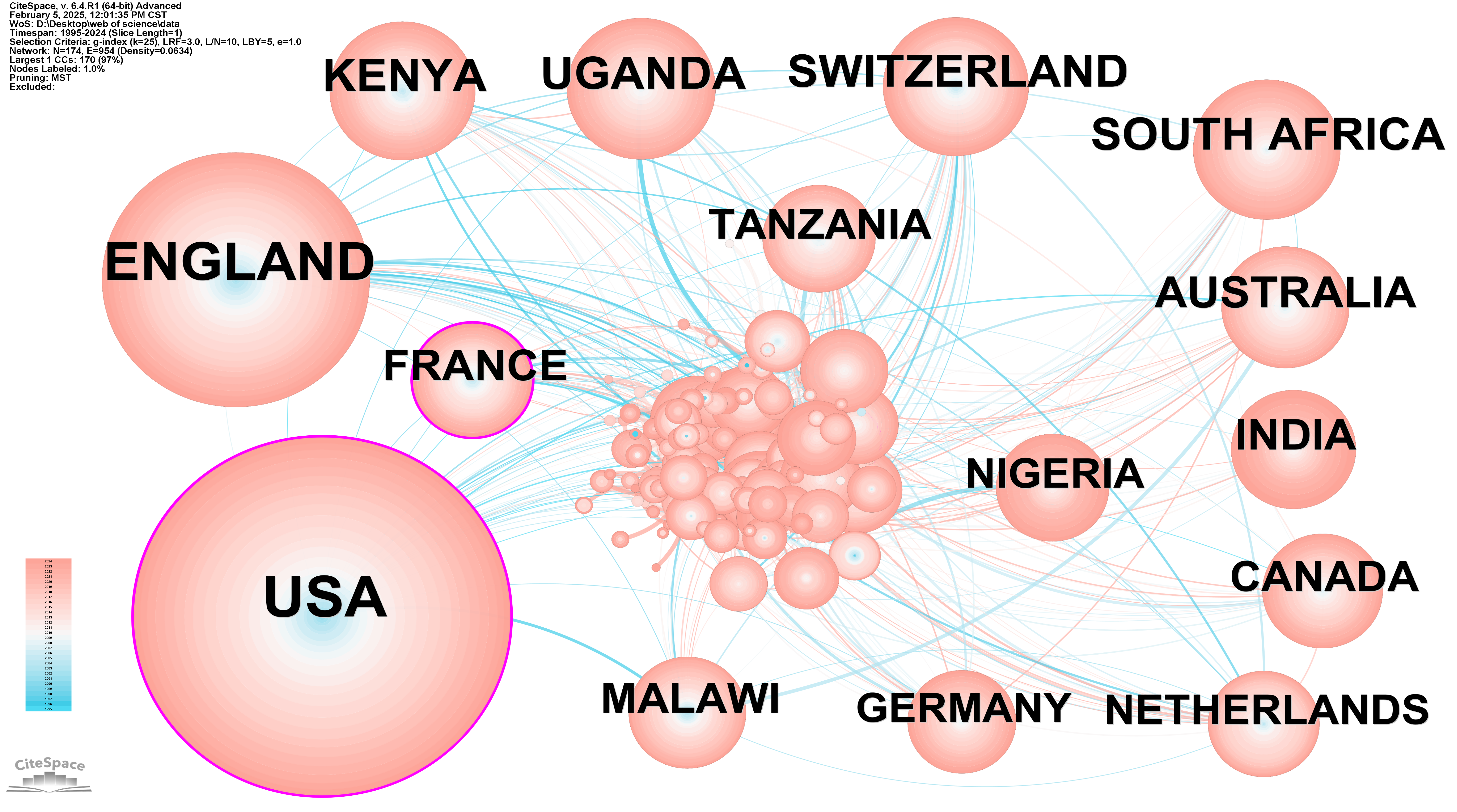

Supplement: Supplementary file 2 [file Image_1.PNG]

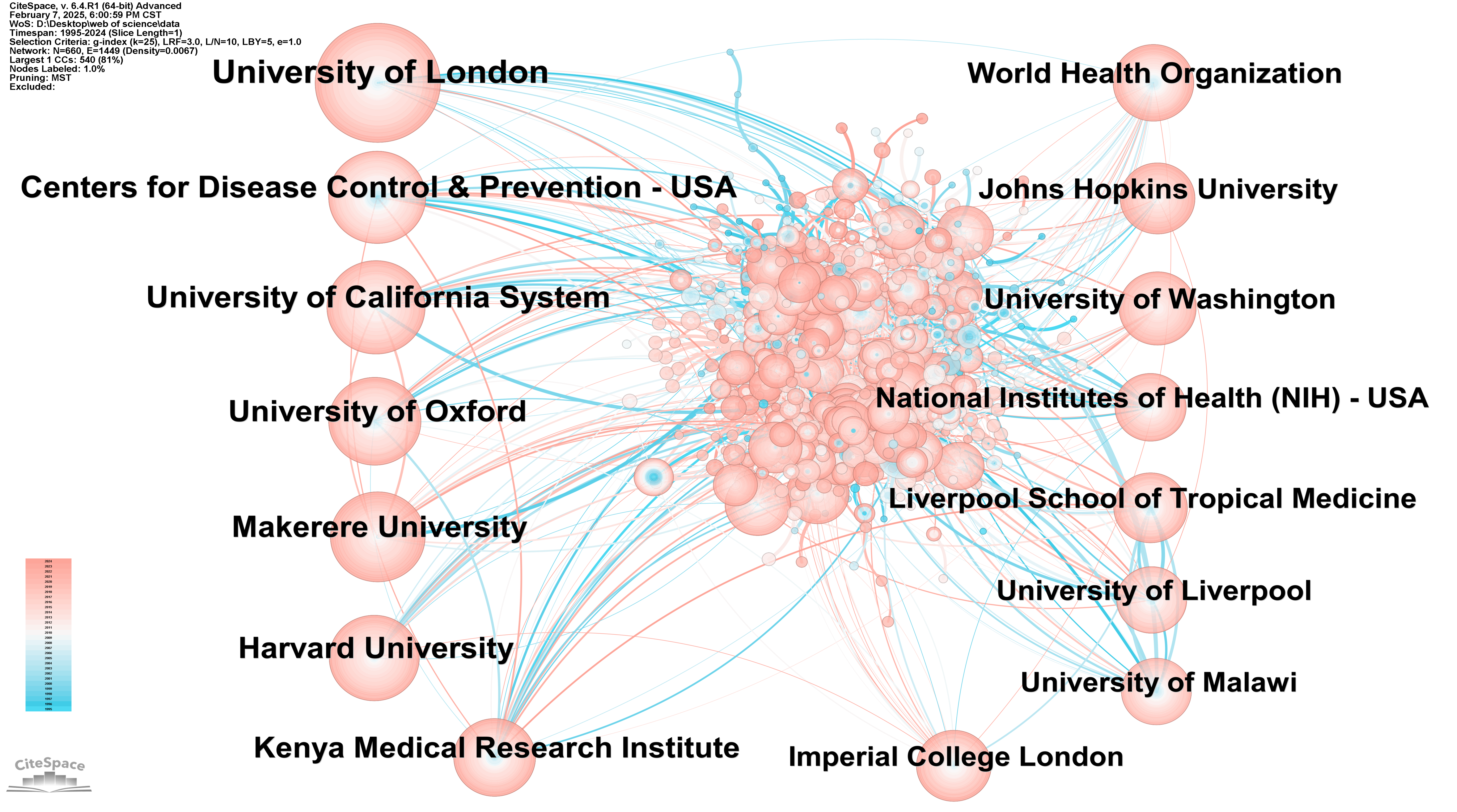

Supplement: Supplementary file 3 [file Image_2.PNG]

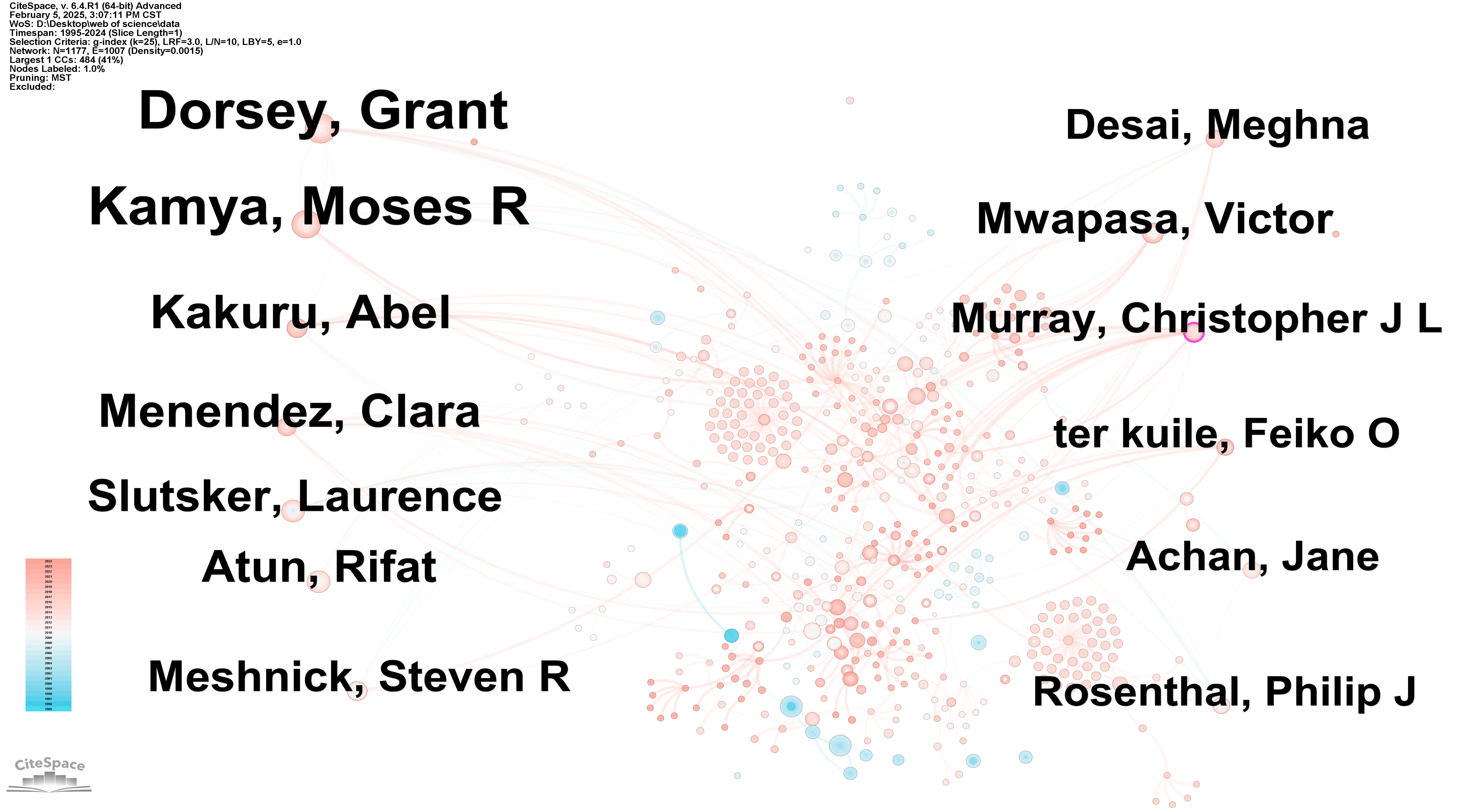

Supplement: Supplementary file 4 [file Image_3.PNG]

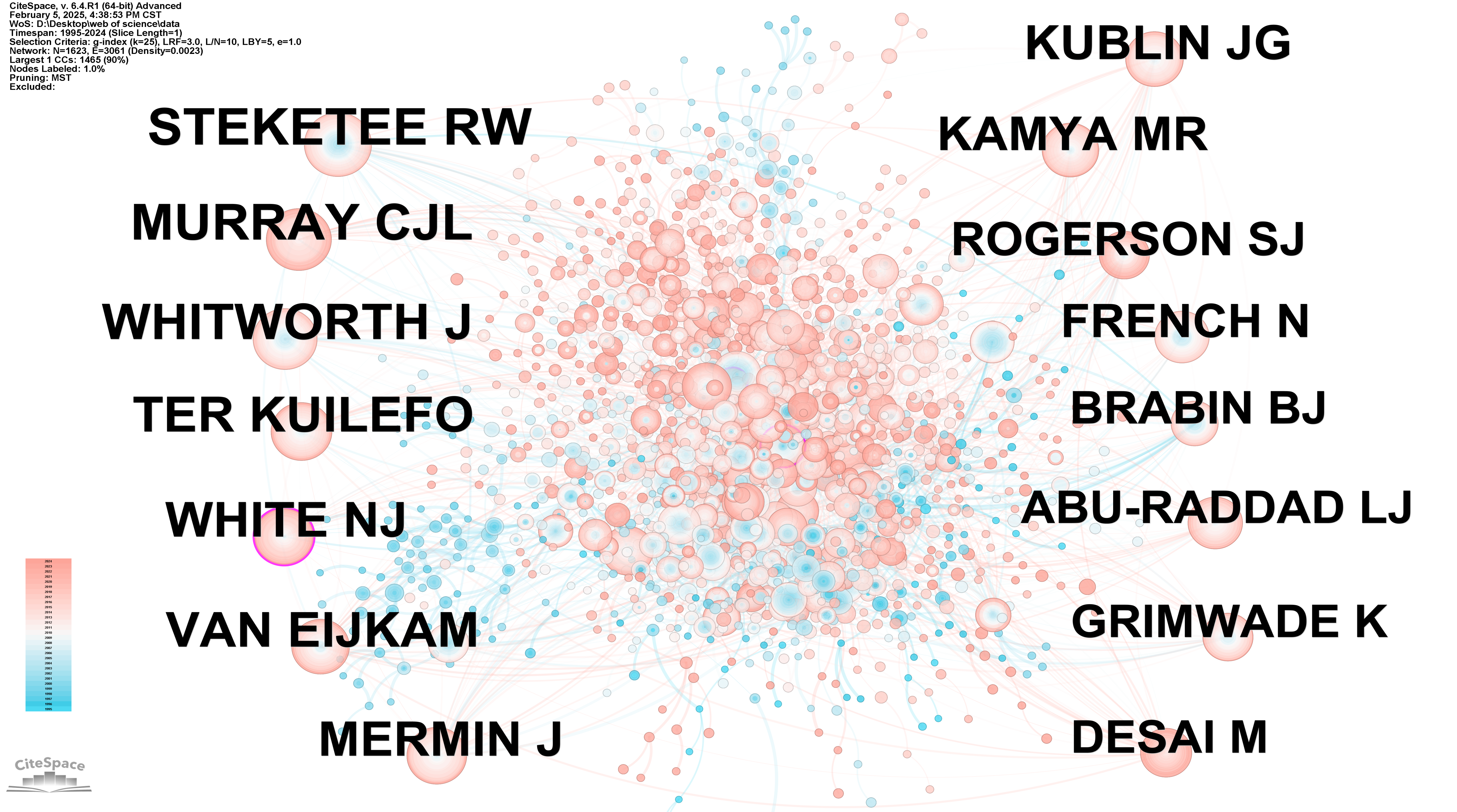

Supplement: Supplementary file 5 [file Image_4.PNG]

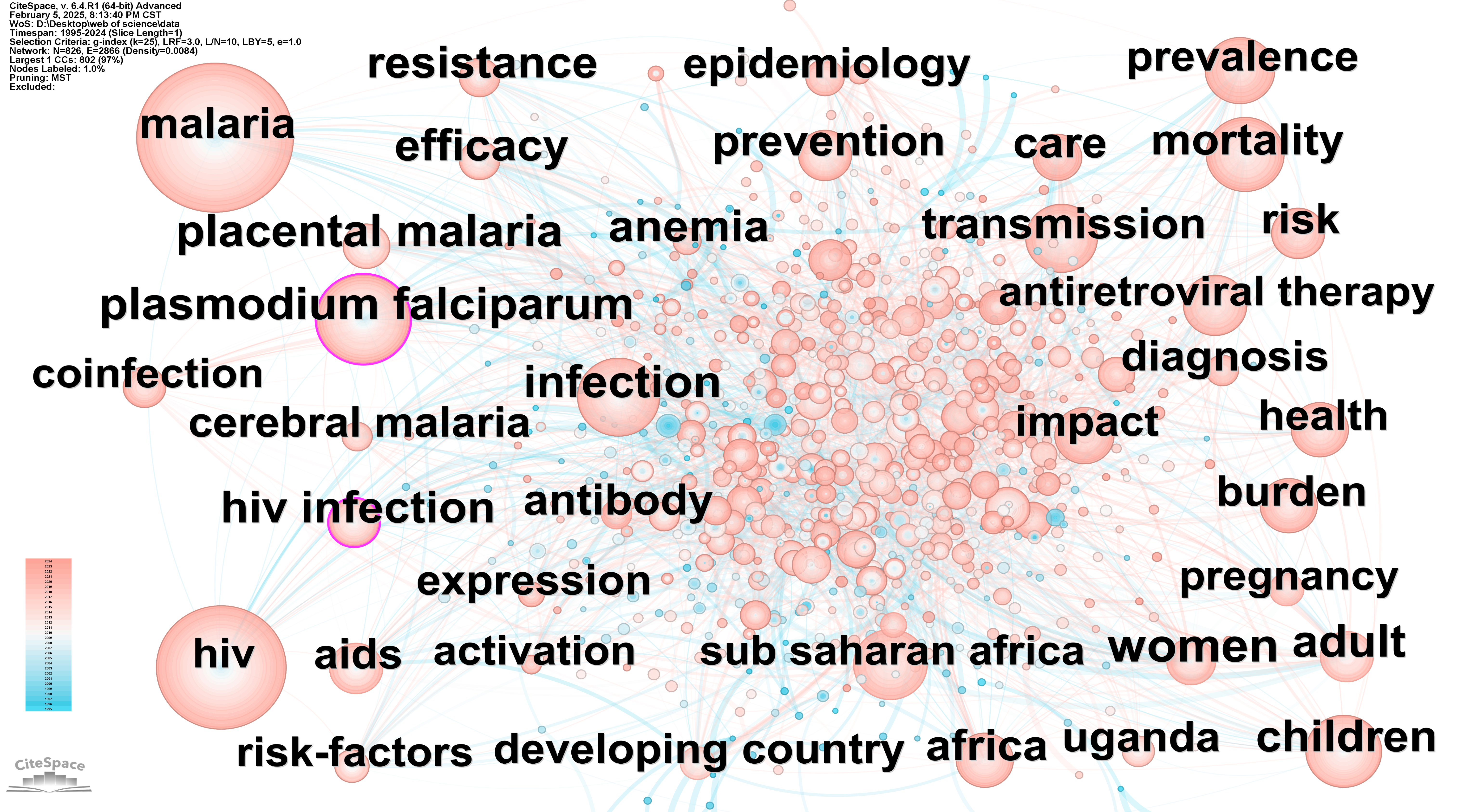

Supplement: Supplementary file 6 [file Image_5.PNG]

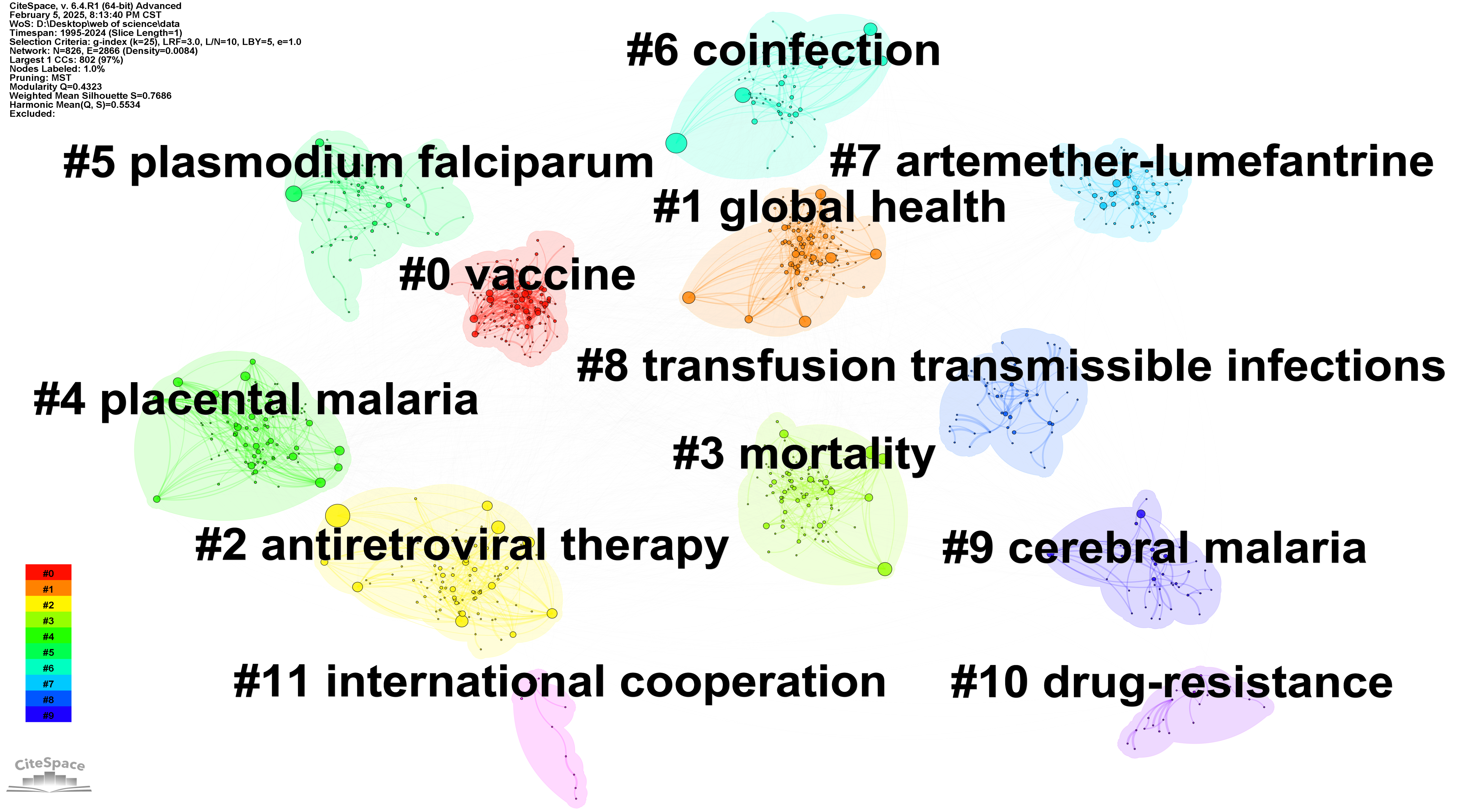

Supplement: Supplementary file 7 [file Image_6.PNG]

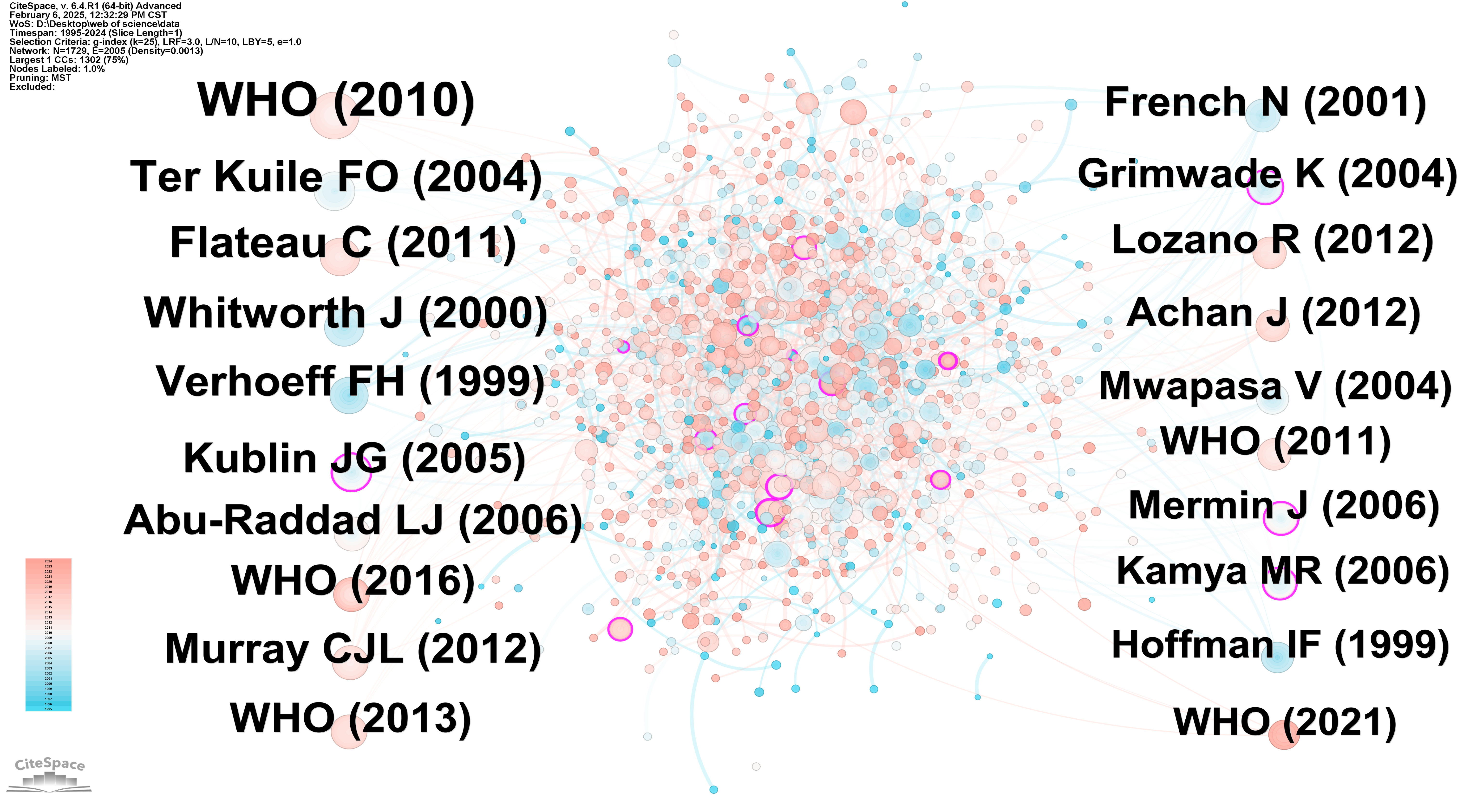

Supplement: Supplementary file 8 [file Image_7.PNG]

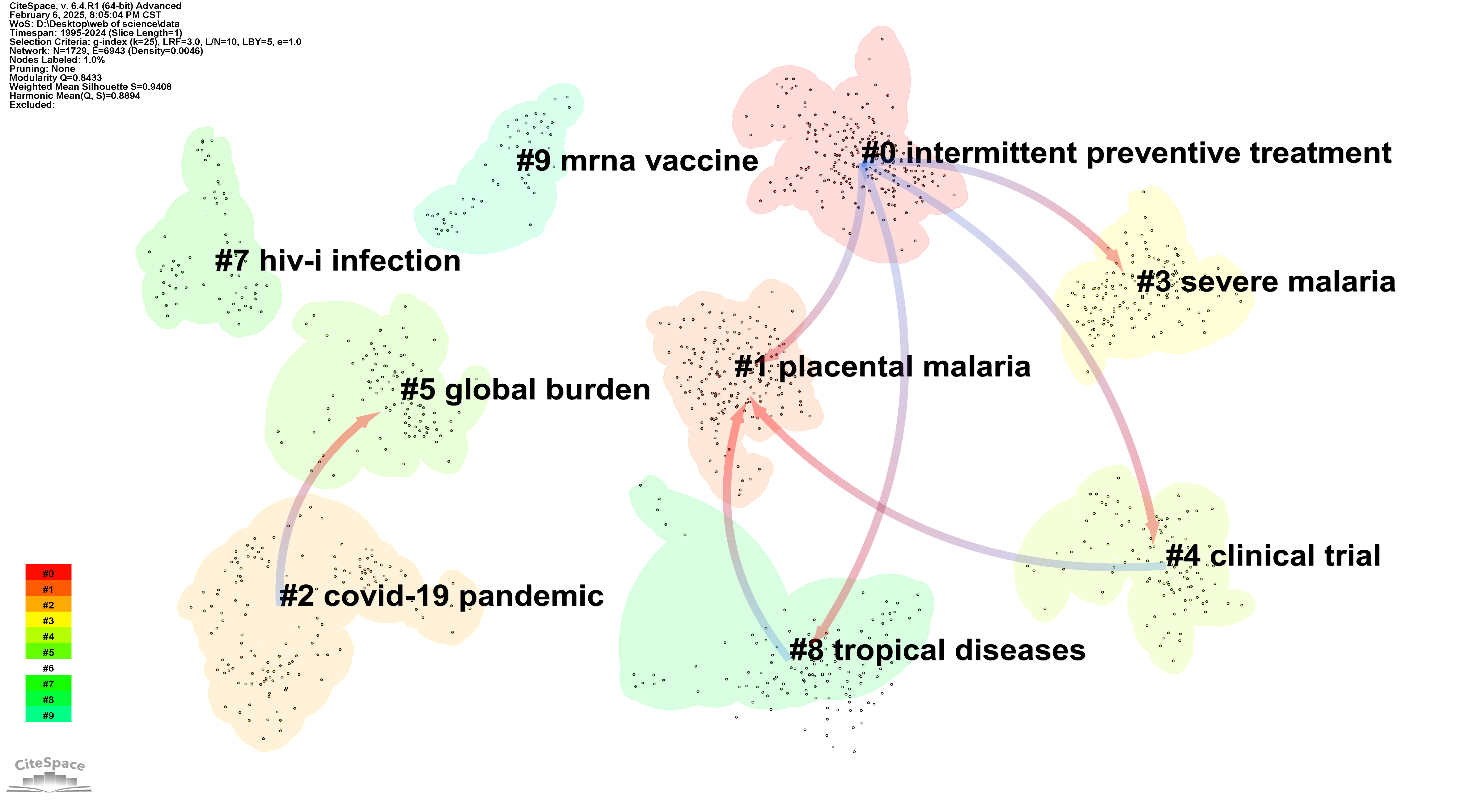

Supplement: Supplementary file 9 [file Image_8.PNG]
