# Supplementary material for: Unraveling the HIV-malaria interactions: a bibliometric analysis of global research trends and emerging insights
Source: Front Microbiol. 2025 Aug 29;16:1622769. doi: 10.3389/fmicb.2025.1622769 (PMC12426043; doi:10.3389/fmicb.2025.1622769)
Supplement: Supplementary file 10 [file Table_1.DOCX]

(TS= "Malaria" OR TS= "Infections, Plasmodium" OR TS= "Infection, Plasmodium" OR TS= "Plasmodium Infection" OR TS= "Plasmodium Infections" OR TS= "Paludism" OR TS= "Remittent Fever" OR TS= "Fever, Remittent" OR TS= "Marsh Fever" OR TS= "Fever, Marsh") **AND** (TS= "HIV" OR TS= "HTLV-III" OR TS= "Human Immunodeficiency Virus" OR TS= "Immunodeficiency Virus, Human" OR TS= "Immunodeficiency Viruses, Human" OR TS= "Virus, Human Immunodeficiency" OR TS= "Viruses, Human Immunodeficiency" OR TS= "Human Immunodeficiency Viruses" OR TS= "Human T Cell Lymphotropic Virus Type III" OR TS= "Human T-Cell Lymphotropic Virus Type III" OR TS= "Human T-Cell Leukemia Virus Type III" OR TS= "Human T Cell Leukemia Virus Type III" OR TS= "LAV-HTLV-III" OR TS= "Lymphadenopathy-Associated Virus" OR TS= "Lymphadenopathy Associated Virus" OR TS= "Lymphadenopathy-Associated Viruses" OR TS= "Viruses, Lymphadenopathy-Associated" OR TS= "Human T Lymphotropic Virus Type III" OR TS= "Human T-Lymphotropic Virus Type III" OR TS= "AIDS Virus" OR TS= "AIDS Viruses" OR TS= "Virus, AIDS" OR TS= "Viruses, AIDS" OR TS= "Acquired Immune Deficiency Syndrome Virus" OR TS= "Acquired Immunodeficiency Syndrome Virus" OR TS= "Acquired Immunodeficiency Syndrome" OR TS= "AIDS" OR TS= "Immunodeficiency Syndrome, Acquired" OR TS= "Acquired Immunodeficiency Syndromes" OR TS= "Immunodeficiency Syndromes, Acquired" OR TS= "Syndrome, Acquired Immunodeficiency" OR TS= "Syndromes, Acquired Immunodeficiency" OR TS= "Acquired Immune Deficiency Syndrome" OR TS= "Acquired Immuno-Deficiency Syndrome" OR TS= "Acquired Immuno Deficiency Syndrome" OR TS= "Acquired Immuno-Deficiency Syndromes" OR TS= "Immuno-Deficiency Syndrome, Acquired" OR TS= "Immuno-Deficiency Syndromes, Acquired" OR TS= "Syndrome, Acquired Immuno-Deficiency" OR TS= "Syndromes, Acquired Immuno-Deficiency" OR TS= "Immunologic Deficiency Syndrome, Acquired")
